# Methods Matter: A Comparative Review of Health Risk Assessments for Ambient Air Pollution in Switzerland

**DOI:** 10.3389/phrs.2022.1604431

**Published:** 2022-04-06

**Authors:** Alberto Castro, Martin Röösli, Kees de Hoogh, Ron Kappeler, Meltem Kutlar Joss, Danielle Vienneau, Nino Künzli

**Affiliations:** ^1^ University of Basel, Basel, Switzerland; ^2^ Swiss Tropical and Public Health Institute (Swiss TPH), Basel, Switzerland

**Keywords:** Switzerland, air pollution, burden of disease, particulate matter, health risk assessment, health impact assessment, mortality

## Abstract

**Objectives:** Air pollution health risk assessments (AP-HRAs) provide a method to quantify health effects for entire populations. In Switzerland, AP-HRAs are included in Swiss assessments for Transport Externalities (STEs), ordered by public authorities since the 1990s. This study aimed to describe the differences among national and international AP-HRAs for Switzerland.

**Methods:** We compared input data, approaches and results across AP-HRAs over time. Results and input data for each AP-HRA were expressed as a ratio compared to the most recent STE (in most cases STE-2010).

**Results:** Substantial variation across AP-HRAs was found. For all-cause adult mortality attributed to particulate matter (the most frequent outcome-pollutant pair), the ratio in HRAs oscillated from 0.40 to 2.09 (times the STE-2010 value). Regarding input data, the ratio ranged from 0.69 to 1.26 for population exposure, from 0 to 1.81 for counterfactual scenario, from 0.96 to 1.13 for concentration-response function and from 1.03 to 1.13 for baseline health data.

**Conclusion:** This study demonstrates that methods matter for AP-HRAs. Transparent and possibly standardized reporting of key input data and assumptions should be promoted to facilitate comparison of AP-HRAs.

## Graphical Abstract

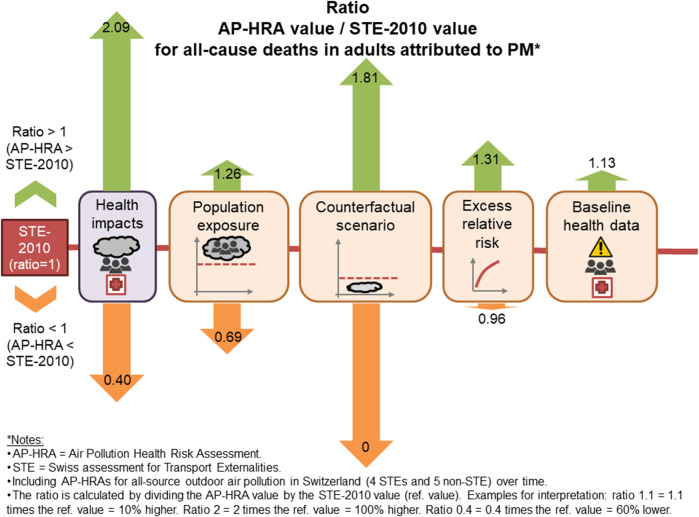

## Introduction

Ambient (outdoor) air pollution causes health effects both in the short- and the long-term, as summarized in many reviews [1]. These health impacts in an entire population can be estimated using a health risk assessment (HRA), which has been defined as “the scientific evaluation of potential adverse health effects resulting from human exposure to a particular hazard” [2]. The concept of health impact assessment is different involving “a combination of procedures, methods and tools” and focusing on a specific “policy, program or project” [3, 5], but the term has also been used interchangeably to refer to HRAs [1]. Particulate matter (PM) up to 2.5 or 10 μm in diameter (PM_2.5_ or PM_10_, respectively), ozone (O_3_) and nitrogen dioxide (NO_2_) are examples of harmful pollutants included in air pollution HRAs (AP-HRAs) [6].

The Swiss government was the first in the world to call for comprehensive assessment studies of environmental effects of heavy duty traffic to integrate the external costs of road transport into the road pricing policy [7], and thus at the interface between health impact assessment and HRA. These studies are abbreviated as STE (Swiss assessment for Transport Externalities). Given first findings of the Swiss SAPALDIA study on health effects of ambient air pollution [8], the Swiss government asked for the inclusion of traffic-related air pollution into STEs. Updates of the STEs were then carried out regularly. This initiative triggered the development of methods to derive the impact of short- and long-term ambient air pollution [9] and to estimate the health burden of traffic related air pollution in Switzerland [10]. Based on a further initiative of the Swiss Government, the first multi-country AP-HRA was conducted in collaboration with France and Austria [11]. This contributed to the inclusion of ambient air pollution into the GBD [12] and later to the development of international methodological standards for AP-HRAs through the project “Health Risks of Air Pollution in Europe” (HRAPIE) [13]. Further updates of the STEs were carried out for 2000 [14], 2005 [15], 2010 [16], 2015 [17] and 2017 [18] and 2018 [19]. In parallel to these Swiss national initiatives, a range of international AP-HRA studies, e.g., the Global Burden of Disease (GBD) [4, 20–22], have included specific estimations of health impacts attributed to air pollution in Switzerland.

Although AP-HRAs usually get high media attention, the ongoing development of AP-HRA methods are rarely addressed and comparison across AP-HRAs are scarce. The work of Malmqvist, Oudin [23] and Evangelopoulos, Perez-Velasco [24] are recent exceptions. As shown in these reviews, methodological approaches for quantifying health impacts and their input data may vary among AP-HRAs and strongly determine their results. The most relevant input data are the following: the population exposure; the counterfactual scenario (i.e., the minimum concentration considered in the AP-HRA to derive the overall impact); the concentration-response function (CRF, usually derived from a meta-analyses of epidemiological studies); and the baseline health data (i.e., prevalence or incidence of the disease data among the population at risk).

The ever-growing number of AP-HRAs for Switzerland proposing different health estimates poses a communication challenge for public authorities. Thus, this paper had as overall goal to identify differences between AP-HRAs of STEs and other AP-HRAs with specific results for Switzerland as well as the reasons behind these differences. To achieve this goal, the following two objectives were pursued: 1) to scrutinize all national and international AP-HRAs assessing health impacts of ambient air pollution for Switzerland, and 2) to compare them with the most recent STE in terms of assessed health impacts and their input data (namely the population exposure, counterfactual scenario, CRF and baseline health data).

## Methods

Out of all published STEs, we selected only those assessing health impacts for ambient air pollution from all sources, i.e., not exclusively for transport related air pollution. Exceptionally, we selected STE-1993, which only showed transport-related health impacts, because it stated that the transport-related exposure represented on average 40% of the total exposure. Therefore, we converted the transport-related health impacts into all source impacts by dividing by 0.4. This conversion implicitly assumed a zero counterfactual scenario to express the share of the total burden attributable to transport alone.

To find further AP-HRAs beyond the governmental STEs, we carried out a literature search with specific search terms using Google Search to capture not only scientific but also grey literature. These additional AP-HRAs had to meet the following inclusion criteria:

• Assessment of health impact from ambient air pollution from all sources of air pollution including burden of disease studies targeted to air pollution such as GBD
• Separate results for each pollutant and for each (present or past) year
• Specific results for Switzerland including the whole population or a large well defined subset of the country
• Original assessment (i.e., not only re-using results of other AP-HRAs)
• Most updated version, in case of multiple published AP-HRAs with the same authors, for the same region and with overlapping years of analysis.

Supplementary Material S1 reports information about the literature excluded from this comparison because of not meeting the inclusion criteria, the specific search terms, and the PRISMA flow chart.

Among the selected AP-HRAs, we compiled health impact estimates for all available years as well as the input data including population exposure, counterfactual scenarios, CRFs and baseline health data. For an overview of the most recent health impact estimations for Switzerland, we prioritized most recent STEs over other AP-HRAs; and if no STE was available, we used the most recent AP-HRAs. For the comparison between STEs and HRAs, we focused on the pollutant with the highest health impacts and selected the first and last year in case of AP-HRAs with time series (i.e., multiple years). We only considered health outcomes assessed by at least two AP-HRAs, being one of them a STE. To analyze the variability of health impacts and input data the following steps were applied. First, the AP-HRA results were normalized by calculating the impact per 100,000 persons (all ages) to remove the effect of population growth. PM_2.5_ data were converted into PM_10_ with the assumption that PM_2.5_ accounts for 73.5% of PM_10_ [26]. Next, we calculated the ratio of the AP-HRA value (numerator) to the reference STE (denominator) to quantify the heterogeneity. Thus, a ratio >1 indicates an AP-HRA with a larger value than the most recent STE and a ratio <1 a lower value. The ratios were used for comparisons across AP-HRAs and across input data (the latter building ranges defined by the minimum and maximal ratio).

We performed the data analysis and visualization in R 4.0.3 [27] using the package tidyverse 1.3.0 [28].

Further details on the methods of this paper, including population data for the normalization, equations for re-scaling as well as data preparation and assumptions for collected data, are available in the Supplementary Material S1.

## Results

### Health Impacts

Table 1 presents the AP-HRAs that we selected for the comparison. Table 2 shows an overview of the most recent health impacts attributed to PM (which refers to PM_2.5_ and/or PM_10_), O_3_ and NO_2_ in Switzerland from the STEs or, if not available, from other AP-HRAs. STE-2010 covered the majority of health endpoints ever assessed by STEs. STEs exclusively considered PM in the assessment, while some of the other AP-HRAs included O_3_ and/or NO_2_. All-cause mortality attributed to O_3_ and NO_2_ exposure was much lower than to PM in Switzerland. For instance the number of all-cause premature deaths in adults attributed to PM was 2,587 according to STE-2010, while it was 247 for all ages for O_3_ according to GBD-2019, and 270 for NO_2_ according to EEA-2018. We confirmed the large differences among pollutants in an additional analysis by analyzing all AP-HRAs covering multiple pollutants in the most recent overlapping year (2015) and by normalizing by population (see Supplementary Material S2). Since STEs exclusively assessed health impacts attributed to PM and the health impacts attributed to O_3_ and NO_2_ were considerably lower, further comparisons of this paper focus on PM only.

**TABLE 1:** Main features of the selected air pollution health risk assessments (Switzerland 2021).

Short name of the AP-HRA study	Year of analysis^a^	Swiss Area^b^	Types of outcomes	Pollutants^c^	Goal	Source
**Studies designed for Switzerland**						
STE	1993, 1996, 2000, 2005, 2010	National	• Mortality	PM_10_	External cost of transport in Switzerland	STE reports for 1993 [7], 1996 [11, 47], 2000 [14], 2005 [15] and 2010 [16]
			• Morbidity			
FCAH	2010	National	• Mortality	PM_10_	Comparison of epidemiological and toxicological approaches	Study ordered by the Swiss Federal Commission for Air Hygiene (FCAH) [26]
**International studies including results for Switzerland**						
GBD	1990–2019	National	• Mortality	PM_2.5_, O_3_	Burden of Disease calculation at global level (including air pollution among other risks)	Assessment of the GBD project in 2019, which includes multiple risk factors (being ambient particulate matter and ozone two of them). The results are stratified by risk factor, country, sex, disease and age. Data can be filtered and downloaded from an online tool [48]. The concentration and CRF data as well as the scientific paper explaining the methodology are published separately [4, 49, 50]
			• Morbidity			
			• Mixed			
EEA	2009,	National	• Mortality	PM_2.5_, O_3_, NO_2_ (in 2011 PM_2.5_, O_3_	Health impacts of air pollution in Europe	European Air Quality Reports of EEA [51-59]. The detailed description of the EEA methodology was published elsewhere [30, 59]
	2011–2018					
WHO	2012, 2016	National	• Mortality	PM_2.5_	Worldwide burden of disease calculation for ambient air pollution	WHO report for 2012, showing specific results by country [31]. An update for 2016 was available as online database [60]. The counterfactual scenario of WHO-2016 was published elsewhere [24]
			• Mixed			
CITIES	2015	10 largest cities	• Mortality	PM_2.5_, NO_2_	Health impacts of air pollution in European urban areas	AP-HRA ordered by the Spanish Ministry of Science and Innovation, which covers 1,000 urban areas in Europe for 2015 [39]

Abbreviations: AP-HRA, air pollution health risk assessment; STE, Swiss assessment for transport externalities; EEA, European Environment Agency; FCAH, Federal Commission for Air Hygiene; GBD, Global Burden of Disease; WHO, World Health Organization. CITIES = HIA for air pollution in around 1000 European urban areas. The short name was given by the authors of this paper.

a Single assessment for each year of analysis, except for GBD, which assessed in 2019 the whole time series 1990–2019, and EEA, which included the assessment of both 2009 and 2018 in the same report from 2020.

b CITIES covers the greater cities of Zurich, Geneva, Basel, Bern, Lausanne, Luzern and Lugano as well as the cities of Winterthur, St. Gallen and Biel/Bienne.

c Health impacts of NO_2_ were estimated in STE-1993, but it was not shown in the final results (but in some kind of Appendix).

**TABLE 2:** Overview of absolute annual health impacts attributed to exposure to air pollution in Switzerland by pollutant (Switzerland 2021).

Mortality vs. Morbidity	Type of impact	Outcome disease	Population group^a^	Absolute number of cases per year^b^	AP-HRA—year of analysis^c^
**PM_10_/PM_2.5_**					
Mortality	Premature deaths	All causes	Adults	2,827	STE-2010
			Infants	13	STE-2010
			Workers	335	STE-2010
		Lung cancer	Adults	311	STE-2000
	Working YLLs	All causes	Adults	2,767	STE-2010
			Infants	346	STE-2010
	YLLs	All causes	Adults	28,138	STE-2010
			Infants	753	STE-2010
Morbidity	Attacks	Asthma	Adults	3,500,000	STE-1993
			Children	44,943	STE-2010
	Attacks (person-days)	Asthma	Adults	107,545	STE-2010
	Cases (incidence)	Acute bronchitis	Children	77,500	STE-1993
		Chronic bronchitis	Adults	3,078	STE-2010
	Cases (prevalence)	Acute bronchitis	Children	17,302	STE-2010
		Chronic bronchitis	Adults	55,000	STE-1993
	Hospital admissions	CVD	All	1,138	STE-2010
		RD	All	1,131	STE-2010
	Hospital days	CVD	All	10,940	STE-2010
		RD	All	9,420	STE-2010
	Invalidity cases	Chronic bronchitis	Adults	25	STE-1993
	Medication intake (person-days)	Asthma	Adults	3,750,000	STE-1993
	RADs	All causes	Adults	4,746,089	STE-2010
	Symptom days	RD	All	20,000,000	STE-1993
			Children	60,000	STE-1993
	Work loss days	All causes	Workers	1,138,140	STE-2010
	YLDs	All causes	All	7,196	GBD-2019
Mixed	DALYs	All causes	All	28,207	GBD-2019
**O_3_**					
Mortality	Premature deaths	All causes	All	247	GBD-2019
	YLLs	All causes	All	3,255	GBD-2019
Mixed	DALYs	All causes	All	3,255	GBD-2019
**NO_2_**					
Mortality	Premature deaths	All causes	Adults	270	EEA-2018
	YLLs	All causes	Adults	2,827	EEA-2018

Abbreviations: YLLs, years of life lost; DALYs, disability-adjusted life years; RADs, restricted activity person-days; YLDs, years lived with disability.

a Age ranges of the population groups differ across AP-HRAs. This table shows only aggregated GBD results, which are additionally available by age range, gender and disease.

b Some of the AP-HRAs provide 95% confidence intervals with a lower bound up to 70% lower and upper bounds up to 70% higher than the point estimates presented in this table.

c We estimated the health impacts of STE-1993 by dividing the transport-related impacts by 0.4 because STE-1993 only assessed transport externalities and pointed out that 40% of the total air pollution exposure account for transport.

As shown in Table 3, all population-normalized mortality impacts were lower in STE-2010 than in previous STEs, EEA-2019 and EEA-2009. In contrast, the STE-2010 values were higher than in GBD-2019, WHO-2012 and WHO-2016. Two AP-HRAs, the FCAH and CITIES, include two assessments because they each used two counterfactual scenarios, respectively called high and low. Premature deaths and YLLs, both in adults, were the most assessed health impact across AP-HRAs. The number of premature deaths in adults per 100,000 inhabitants differed from the STE-2010 by a factor of 0.4–2.09, while for YLLs in adults per 100,000 inhabitants this ratio varied from 0.49 to 2.55.

**TABLE 3:** Annual mortality impacts attributed to particulate matter across air pollution health risk assessments, years and counterfactual scenarios expressed as per 100,000 inhabitants (all ages) and ratio in relation to the reference value, i.e., the most recent Swiss assessment for Transport Externalities. The ratio was calculated by dividing the value of the health risk assessment by the reference value (Switzerland 2021).

Type of Impact	Outcome disease	population Group^a^	STE	STE	STE	STE	STE	EEA	EEA	FCAH	FCAH	GBD	GBD	WHO	WHO	CITIES	CITIES
			1993^b^	1996	2000	2005	2010	2009	2018	2010	2010	1990	2019	2012	2016	2015	2015
										Low^c^	High^c^					Low^c^	High^c^
Mortality per 100,000 all-age persons																	
Premature deaths	All causes	Adults	76	47	52		**36**	64	41			53	16	19	26	44	14
		Infants			0.3		**0.2**					0.3	0.1	0.0			
	Lung cancer	Adults			**4**					5	3	7	3	5	3		
Working YLLs	All causes	Adults			74		**36**										
YLLs	All causes	Adults			569	624	**361**	721	459			922	237	327	379	540	177
		Infants			24	26	**10**					23	9	0.4	26		
Ratio in relation to reference value (last STE)^d^																	
Premature deaths	All causes	Adults	2.09	1.29	1.44		**1**	1.75	1.14			1.46	0.44	0.51	0.70	1.20	0.40
		Infants			1.50		**1**					1.50	0.50	0.00			
	Lung cancer	Adults			**1**					1.07	0.77	1.65	0.65	1.19	0.58		
Working YLLs	All causes	Adults			2.07		**1**										
YLLs	All causes	Adults			1.57	1.73	**1**	1.99	1.27			2.55	0.65	0.90	1.05	1.49	0.49
		Infants			2.44	2.68	**1**					2.35	0.90	0.04			

Abbreviations: YLLs, years of life lost. Bold values represent the reference values.

a Age ranges of the population groups differ across AP-HRAs.

b We estimated the health impacts of STE-1993 by dividing the transport-related impacts by 0.4 because STE-1993 only assessed transport externalities and pointed out that 40% of the total air pollution exposure account for transport.

c FCAH and CITIES, include two assessments—respectively called high and low–because they each use a lower and a higher counterfactual scenario.

d Examples for interpretation of the ratio: 1.1 = 1.1 times the ref. value = 10% higher. 2.0 = 2 times the ref. value = 100% higher. 0.4 = 0.4 times the ref. value = 60% lower.

**FIGURE 1:**
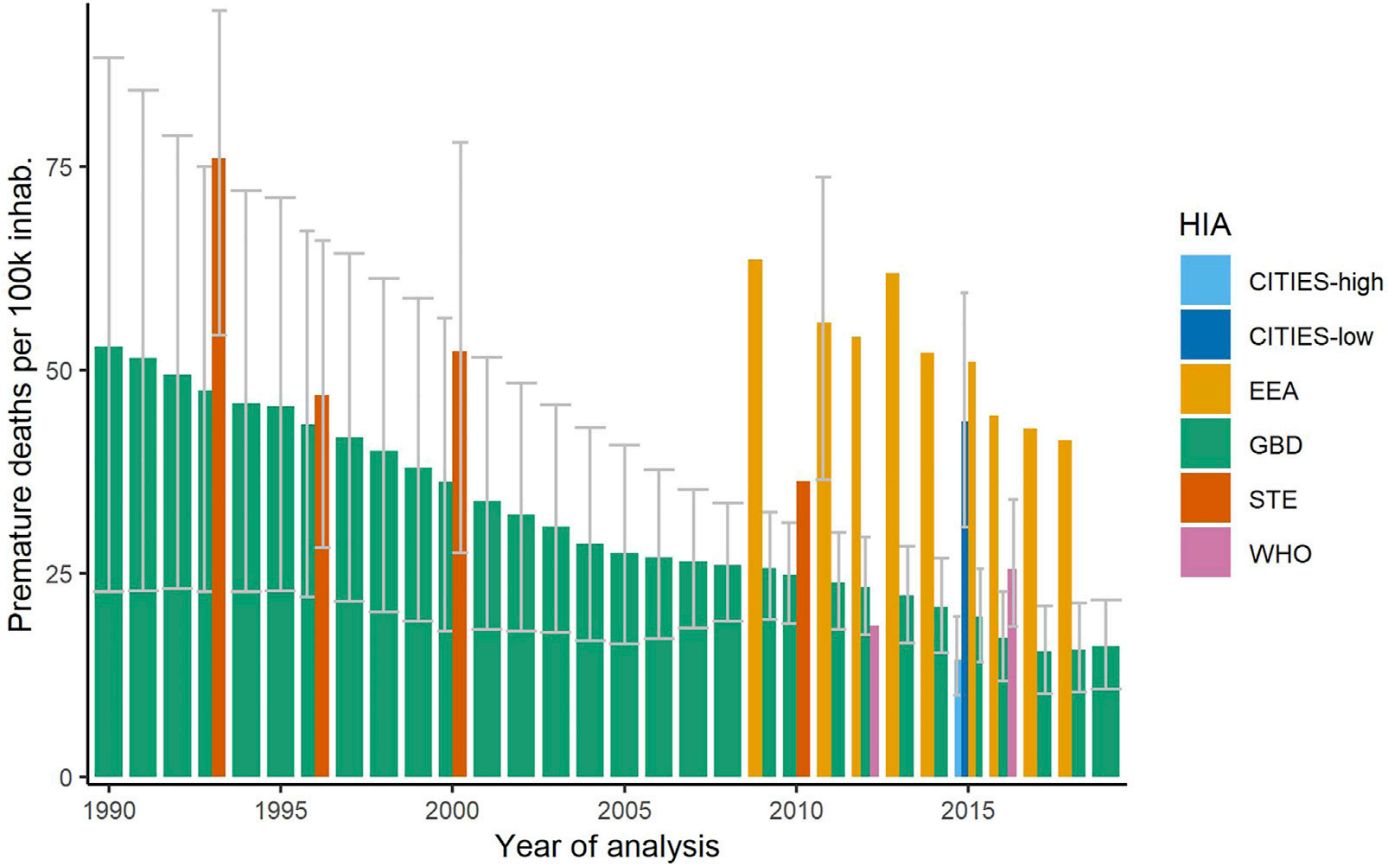
Annual premature deaths per 100,000 persons in adults attributed to particulate matter with 95% confidence interval (if available) (Switzerland 2021).

Figure 1 shows that, as assumed, the first and last year of time series captured the whole heterogeneity of the population-normalized premature deaths in adults over time, when considering the entire GBD and EEA time series (see values in Supplementary Material S2). Only the GBD values for 2017 and 2018 were slightly lower than the value for 2019 (the last year of the time series). Furthermore, population-normalized premature deaths were considerably higher for EEA than for GBD in overlapping years. Regarding YLLs, the differences between EEA and GBD were smaller (see Supplementary Material S2).

The morbidity outcomes of STEs were not assessed in other AP-HRAs, which focus on indicators such as Years Lived with Disabilities (YLDs) and Disability-Adjusted Life Years (DALYs). Comparisons of health impacts, CRFs and baseline health data for morbidity across STEs are available in the Supplementary Material S2.

### Population Exposure

Population exposure refers to the (measured or modeled) air pollution concentration that is assumed to cause health impacts. The population-weighted annual mean exposures estimated in AP-HRAs spanned from 0.69 to 1.26 times the STE-2010 reference value (Table 4).

**TABLE 4:** Annual mean population exposure, counterfactual scenario and difference between both for particulate matter up to 10 μm in diameter across air pollution health risk assessments, years and counterfactual scenarios expressed as a concentration in µg/m_3_ and as a ratio in relation to the reference value, i.e., the most recent Swiss assessment for Transport Externalities. The ratio was calculated by dividing the value of the health risk assessment by the reference value (Switzerland 2021).

Type of Concentration	STE	STE	STE	STE	STE	EEA	EEA	FCAH	FCAH	GBD	GBD	WHO	WHO	CITIES	CITIES
	1993	1996	2000	2005	2010	2009	2018	2010	2010	1990	2019	2012	2016	2015	2015
								Low^a^	High^a^					Low^a^	High^a^
Population-weighted annual mean in μg/m^3^ PM_10_															
Exposure	20.9	21.4	19.1	19.7	**19.4**	19.9	13.3	18	18	24.5	13.5		13.9	17.7	17.7
Counterfactual		7.5	7.5	7.5	**7.5**	0	0	3.3	7.5	5.6	5.6	9.9	5.6	5.0	13.6
Difference		13.9	11.6	12.2	**11.9**	19.9	13.3	14.7	10.5	18.8	7.8		8.2	12.7	4.1
Ratio in relation to reference value (last STE) ^b^															
Exposure	1.08	1.10	0.98	1.02	**1**	1.03	0.69	0.93	0.93	1.26	0.70		0.72	0.91	0.91
Counterfactual		1.00	1.00	1.00	**1**	0	0	0.44	1.00	0.75	0.75	1.32	0.75	0.67	1.81
Difference		1.17	0.97	1.03	**1**	1.67	1.12	1.24	0.88	1.58	0.66		0.69	1.07	0.34

Notes: The PM concentrations were originally expressed as PM_2.5_ instead of as PM_10_ in EEA, GBD and WHO. We re-scaled these concentrations to enable comparability across AP-HRAs. The original values are available in the Supplementary Material. STE-1993 had no counterfactual scenario because it quantified the impact of transport related emissions. Bold values represent the reference values.

^a^ FCAH and CITIES, include two assessments—respectively called high and low–because they each use a lower and a higher counterfactual scenario.

^b^ Examples for interpretation of the ratio: 1.1 = 1.1 times the ref. value = 10% higher. 2.0 = 2 times the ref. value = 100% higher. 0.4 = 0.4 times the ref. value = 60% lower.

The population-weighted exposure used in STE-2010 was 19.4 μg/m^3^ PM_10,_ while it was 13.3 and 13.5 for GBD-2019 and EEA-2018, respectively (the AP-HRAs with the largest differences). The population-weighted mean exposure estimated in AP-HRAs for Switzerland has decreased (with few exceptions) over time and the values slightly differed in overlapping years across AP-HRAs, as an additional analysis including the entire EEA and GBD time series showed (see Supplementary Material S2).

Population exposure data, including conversion from PM_2.5_ to PM_10_, are available in the Supplementary Material.

### Counterfactual Scenario

The counterfactual scenario refers to the lowest concentration used for comparison with the respective population-weighted annual mean exposure. Health impacts below this cut-off are excluded from the assessment either because they are considered to have insufficient scientific evidence or deemed not relevant for the AP-HRA (e.g., to exclude natural air pollution sources). The STEs used the term “reference concentration” for the “counterfactual scenario”.

STE-2010, as previous STEs, chose 7.5 μg/m^3^ PM_10_ as counterfactual scenario, while other AP-HRAs used values from 0 to 81% higher, i.e., ratio 0 to 1.81 (Table 4). STE-1993 had no counterfactual scenario because it quantified the impact of all transport related emissions. The other STEs chose 7.5 μg/m^3^ PM_10_ arguing that, although there was no evidence of a threshold, the existing literature included only populations with at least 5–10 μg/m^3^ annual mean concentrations. Thus, the average was used as counterfactual scenario. The FCAH considered a “high” counterfactual scenario assuming 7.5 μg/m^3^ PM_10_ for comparability with STEs and a “low” scenario assuming 3.3 μg/m^3^ PM_10_ based on a recent publication which derived a CRF down to this level [29]. CITIES also used two scenarios. The high scenario corresponded to the WHO Air Quality Guideline value (10 μg/m^3^ PM_2.5,_ i.e., 13.6 μg/m^3^ PM_10_) and the low scenario corresponded to the lowest measured exposure in the considered European cities (3.7 μg/m^3^ PM_2.5_, i.e., 5 μg/m^3^ PM_10_). EEA assumed zero exposure as counterfactual scenario arguing that the HRAPIE report recommends “the quantification of health impacts at all concentrations” [30]. WHO and GBD counterfactual scenarios were expressed as a uniform distribution from 2.4 to 5.9 μg/m^3^ PM_2.5_ for GBD and WHO-2016 as well as from 5.9 to 8.7 μg/m^3^ PM_2.5_ for WHO-2012. These bounds referred to the minimum and 5th percentiles of air pollution concentrations observed in relevant cohort studies [31], which provided “the uncertainty regarding the level at which the scientific evidence was consistent with adverse effects of exposure” [4]. We calculated the average of these bounds (and re-scaled from PM_2.5_ to PM_10_), to enable comparisons with the other AP-HRAs, resulting in 5.6 μg/m^3^ PM_10_ for GBD and WHO-2016 and 9.9 μg/m^3^ PM_10_ for WHO-2012.

In AP-HRAs, health impacts are derived on the basis of the difference between the population-weighted mean exposure and the counterfactual scenario. This difference in the selected AP-HRAs was 0.34–1.67 times the STE-2010 value (Table 4). More specifically, this difference was lower for STE-2000, GBD-2019, WHO-2016 as well as in the high scenario of FCAH-2010 and CITIES-2015 than for STE-2010.

### Concentration-Response Functions

Table 5 shows the CRFs for mortality outcomes in form of mean excess relative risk (relative risk minus one) per 10 μg/m^3^ PM_10_ and the ratios in relation to the reference value, i.e., the most recent STE. The ratio for all-cause mortality in adults (the most assessed health impact) ranged from 0.96 to 1.31.

**TABLE 5:** Mean excess relative risk of mortality impacts expressed as per 10 μg/m^3^ particulate matter up to 10 μm in diameter, baseline health data across health impact assessments and years expressed as per 100,000 inhabitants (all ages) and as a ratio in relation to the reference value, i.e., the most recent Swiss assessment for Transport Externalities. The ratio was calculated by dividing the value of the health risk assessment by the reference value (Switzerland 2021).

Input Data	Outcome disease for mortality	Population group^a^	STE	STE	STE	STE	EEA	FCAH	GBD	WHO	CITIES
			1993	1996	2000	2010	2009–2018	2010	1990–2019	2012–2016	2015
Concentration-response function expressed as excess relative risk	Mean per 10 μg/m^3^ PM_10_										
	All causes	Adults	0.044	0.043	0.059	**0.045**	0.045		Seven causes	Five causes	0.051
		Infants			0.056	**0.040**					
	Lung cancer	Adults			**0.106**			0.060	0.112		
	Ratio in relation to reference value (last STE)^b^										
	All causes	Adults	0.98	0.96	1.31	**1**	1				1.13
		Infants			1.40	**1**					
	Lung cancer	Adults			**1**			0.57	1.06		
Baseline health data	Value per 100,000 all-age persons										
	All causes	Adults	799	828	809	**735**	Not provided		Not provided	Not provided	759
		Infants			5	**4**					
	Lung cancer	Adults			**39**			55			
	Ratio in relation to reference value (last STE)^b^										
	All causes	Adults	1.09	1.13	1.10	**1**					1.03
		Infants			1.29	**1**					
	Lung cancer	Adults			**1**			1.40			

Note: STE-2005 used the CRFs of 2000 and did not provided baseline health data. Bold values represent the reference values.

a Age ranges of the population groups differ across AP-HRAs.

b Examples for interpretation of the ratio: 1.1 = 1.1 times the ref. value = 10% higher. 2.0 = 2 times the ref. value = 100% higher. 0.4 = 0.4 times the ref. value = 60% lower.

Regarding all-cause mortality in adults, EEA used the same CRF as STE-2010, from the HRAPIE recommendations [13], while CITIES used a higher CRF based on a more recent WHO meeting report [32] and STE-2000 a lower CRF based on an own meta-analysis of three studies [33–35]. For lung cancer mortality, STE-2000 carried out a purpose-designed meta-analysis among the same three studies as for all-cause mortality, while FCAH used a meta-analysis by Huang, Pan [36] after considering eight alternatives. CRFs in form of relative risk, before and after re-scaling from PM_2.5_ to PM_10_, as well as the excess relative risk and the ratios of morbidity outcomes are available in the Supplementary Material.

Regarding the methodological approaches used for health impact quantification, we found differences across AP-HRAs, as described below, in terms of definition of mortality in the CRFs (all-cause vs. cause-specific mortality), shape of CRFs, Population Attributable Fractions (PAF) and quantification of mortality estimates.

Both the GBD and WHO did not use a single all-cause CRF (as in the other AP-HRAs); rather multiple cause-specific estimates were used and the impacts aggregated to obtain the all-cause mortality estimates. WHO considered five causes of deaths (lower respiratory disease, chronic obstructive pulmonary disease, ischemic heart disease, lung cancer and stroke), and the GBD seven (additionally considering diabetes and adverse birth outcomes). Moreover, STE-2010, EEA, GBD and WHO obtained health impacts stratified by sex and age, while CITIES stratified by age. In a later step, stratified impacts were aggregated to obtain all-cause impacts.

STE-1996 and STE-2000 assumed a linear CRF, while the others used a log-linear function. In 2010 the GBD developed the so-called “integrated exposure-response risk functions” based on computer simulations [4, 37]. These functions have been updated over time. WHO-2012 used the 2013 version and WHO-2016 the one from 2015, which were superseded by the most recent update of GBD 2019 (included in our comparison).

Depending on the AP-HRA, the PAF can be calculated assuming a single population-weighted exposure level for the whole country or by smaller spatial units of analysis such as regions. GBD, WHO and CITIES applied the second approach, calculating the exposure for all ages combined. While the STE-2010 applied the first approach, the population-weighted PM exposures were specifically calculated by population group (children, adults or all).

Instead of the general approaches described above, STE-2010 used life tables (i.e., demographic data containing the probability of death for each age group) to quantify both premature deaths and YLLs. EEA and CITIES only used them for YLLs.

Equations used in the selected AP-HRAs for their approaches are available in the Supplementary Material S2.

### Baseline Health Data

Table 5 shows the mortality baseline health data per 100,000 inhabitants and their ratios calculated as the AP-HRA value divided by the reference value (from most recent STE). The ratio for all-cause mortality in adults (the most assessed outcome) ranged from 1.03 to 1.13.

Looking at differences between specific AP-HRAs, CITIES used a higher baseline all-cause mortality than STE-2010, and FCAH a higher one than STE-2000 for cancer mortality. Baseline health data were not reported in GBD, WHO and EEA studies.

Differences in age ranges in population at risk (based on the age defined in CRFs), together with differences in definition of outcomes and methodologies of data sources, lead to differences in baseline health data. Thus, most AP-HRAs assume age of less than 1 year for infant mortality, while WHO included ages below 5. For adults, most AP-HRAs assume ages of 30 and above, while WHO derived the burden for those above 25 and CITIES above 20 years old.

## Discussion

### Main Findings

The aim of this study was to explore differences between the STEs, especially STE-2010, and other AP-HRAs for Switzerland. Our results indicate that the variation of health impacts obtained across AP-HRAs, and over time can be wide. Indeed, the most frequently assessed outcome-pollutant pair, i.e., the number of premature deaths in adults per 100,000 inhabitants attributed to ambient PM exposure, ranged from 14 to 76 (with STE-2010 reporting 36). Thus, the ratios ranged from 0.4 to 2.09 times the STE-2010 value, which was used as reference.

The divergences in approaches and input data used in the AP-HRAs played a role. Overall, for the above mentioned outcome-pollutant pair, the choice of the counterfactual scenario showed the highest heterogeneity among the input data (ratio from 0 to 1.81) followed by the population exposure (0.69–1.26), the CRF (0.96–1.31) and baseline health data (1.03–1.13).

The values of the counterfactual scenario and the CRF relied on choices of authors of AP-HRAs and available evidence. The choice of the counterfactual scenario was based on the specific assumption related to the goal of the AP-HRAs and supported by literature, while the CRF was chosen among available (or purpose-designed) meta-analyses of multiple epidemiological studies. Sometimes published meta-analyses provided multiple CRFs to select from. For instance, FCAH chose a CRF among nine available CRFs, which differed in terms of source (three were considered), type of PM (PM_2.5_ vs. PM_10_), health endpoint (cases vs. deaths) and geographical scope (worldwide vs. Europe) [26].

Differences in population-weighted exposure appeared not only when comparing different years, but also in the same year across different AP-HRAs. An obvious reason behind these divergences was the different level of resolution of the underlying air pollution exposure models and subsequent aggregation to the population weighted mean exposure. The grid size of these models for PM was 200 m × 200 m for STE-2000, 2005 and 2010 [14–16, 38], 250 m × 250 m for CITIES [39], 1 km × 1 km for EEA [30] and STE-1996 [11], and 0.1° × 0.1° for GBD [4] and WHO [31] which is equivalent to 11 km × 7 km in Europe [23]. Larger grid sizes would smooth the variation in concentrations, minimizing the exposure contrasts. This naturally influences the subsequent population-weighted mean [40], and can be more of an issue for pollutants like NO_2_ that vary over small spatial scales (e.g., decay to background levels within hundreds of meters from roads). Furthermore, modifiable areal unit problems and rounding issues explain further differences in health impacts. The former affect aggregations of point or small scale based measures into larger geographic scales and has no solution [41], while the latter can be minimized by applying a generous and consistent rounding in final results which avoids a claim for pseudo-precision. We identified differences in terms of age ranges considered as population at risk, partly due to age differences in the population used for the derivation of CRFs. The broader the age range, the larger the number of people included in the baseline health data and, consequently, the larger the health impacts. However, the final weight of this issue was limited. For instance, according to the assessment of GBD-2019, the number of all-cause premature deaths in the total population was only 0.62% higher than in the population 20 years and older (as in CITIES) or 25 years and older (as in WHO) and 0.67% higher than for ages of 30 and older (as in EEA and STEs).

EEA mortality impacts were considerably higher than STE-2010 impacts, even in closer years of analysis. For instance, premature deaths in adults were 1.75 times higher for EEA-2009 than for STE-2010. The main reason for such divergence was the choice of the counterfactual scenario (0 instead of 7.5 μg/m^3^ PM_10_). EEA uses the same CRF as in STE-2010 and a similar reference concentration (we did not find EEA baseline health data). STE-2010 applies a different method for quantification of premature deaths (life table approach). However, it may not lead to large differences because when using both the life table approach, for YLLs, the differences in results were even larger.

CITIES mortality impacts relative to the STE-2010 impacts depended on the scenario. Regarding the low scenario, the difference between population exposure and counterfactual scenario was 7% higher in CITIES-2015 than in STE-2010 and the CRF 13% higher (baseline health data were very similar in both). This partly explains the up to 50% higher mortality impacts. Regarding the high scenario, the difference between population exposure and counterfactual scenario in CITIES-2015 was only around one third of the value for STE-2010, which counteracted the effect of the 13% higher CRF and partly explained the lower health impacts. It is worth mentioning that in the comparison CITIES-2015 vs. STE-2010, two opposite effects interplayed. Since CITIES-2015 only cover urban (and therefore more polluted) areas, a higher impact per inhabitant could be expected. On the other hand, since CITIES-2015 assesses the impacts 5 years later, a lower impact could be expected (following the decreasing pollution levels in Switzerland).

Whereas, WHO and GBD mortality (especially in terms of YLLs in adults) were rather similar to STE impacts. This similarity between WHO and GBD was expected because the former was partly based on the methodology of the latter (WHO-2012 on GBD update for 2013 and WHO-2016 on GBD update for 2015). The difference between WHO and GBD in this study was somewhat larger than the one reported in a previous international comparison of AP-HRAs [24]. This was because we compared WHO-2012 and WHO-2016 with the GBD update for 2019 instead of with the GBD update for 2013 and 2015.

### Limitations

Our study is a unique comparison of national and international AP-HRAs for a specific country, which includes rarely available quantitative comparisons of health impacts and the related input data. However, we have to acknowledge that comparability across the selected AP-HRAs was limited in some regards. Firstly, the year of analysis of the selected AP-HRAs rarely overlap. We partly corrected for this by normalizing for population, although this correction did not cover influences of other demographic changes such as in life expectancy or in age distribution. Secondly, CITIES covered the ten largest urban areas in Switzerland, which represent 27% of the Swiss population. Therefore, input data and results may be biased towards rather urban and more polluted areas. Thirdly, we converted the transport-related health impacts of STE-1993 into all-source health impacts by dividing by 0.4 because it states that on average 40% of the total air pollution exposure were caused by transport. However, 40% was just the average, while this value can reach up to 60% in some areas. Fourthly, the CRFs of GBD and WHO are based on multiple causes of death instead on a single CRF, which does not enable a direct comparison. Fifthly, we acknowledge that the literature search for the selection of AP-HRAs was limited. Due to the commercially driven algorithm of Google Search and the small number of results [50] retrieved and screened for eligibility; some AP-HRA might be unintentionally left out of the selection. Finally, baseline health data were not reported in some of the selected AP-HRAs. However, these AP-HRAs used international data sets that rely on national data collections (as the ones used in STEs). Therefore, no large differences are expected among them.

### Implications for Existing and Forthcoming Research

The result of this study was consistent with existing literature. Two previous reviews [23, 24] found that the mortality attributed to ambient air pollution was substantially different across international AP-HRAs. The review of Evangelopoulos, Perez-Velasco [24], comparing international AP-HRAs, the highest number of premature deaths was around 3 times higher than the lowest one, whereas we report a 5-fold range across AP-HRAs for Switzerland (76 vs. 14 deaths in adults per 100,000 inhabitants). Both above-mentioned reviews found similar differences in terms of methodological approaches and input data across AP-HRAs, which may explain the different results, with the exception of the counterfactual scenario in the work of Evangelopoulos, Perez-Velasco [24], with a range rather smaller than in our study.

Given such differences across AP-HRAs, it would be desirable that forthcoming AP-HRAs redouble efforts showing transparently the methodological approach and the input data to enable comparisons. Moreover, a lack of agreement concerning terminology and the corresponding equations have been already documented, e.g., for PAF [42, 43]. A full consistency across AP-HRAs is probably unpractical. However, some agreement in basic assumptions and transparent reporting would increase the comparability across AP-HRAs. International agreements on AP-HRAs, e.g., regarding general guiding principles [44], air quality guidelines (e.g., 45) or updated HRAPIE recommendations [46] would no doubt help to unify criteria.

### Conclusion

Even for low population exposure, health impacts are considerable. AP-HRAs for Switzerland use different methodological approaches and input data, which result in different estimated health impacts for all-cause mortality in adults related to PM ranging from 0.4 to 2.09 times the STE-2010 estimate. The largest differences among input data were found in terms of assumptions for counterfactual scenarios, which was owed to different motivations and goals to conduct a specific AP-HRA (e.g., impact of regulation vs. impact of total air pollution). International cooperation based on consensus decisions, for example under the umbrella of the WHO, and further research is required to develop updated guidelines for the application of AP-HRAs regarding methodology, the choice of input data, and the derivation of counterfactual scenarios. Such international agreement may increase consistency across future AP-HRAs and reduce challenges in terms of communication of results.
