# Supplementary material for: Methods Matter: A Comparative Review of Health Risk Assessments for Ambient Air Pollution in Switzerland
Source: Public Health Rev. 2022 Apr 6;43:1604431. doi: 10.3389/phrs.2022.1604431 (PMC9020261; doi:10.3389/phrs.2022.1604431)
Supplement: Supplementary file 1 [file DataSheet2.PDF]

SUPPLEMENTARY MATERIAL 2: RESULTS

SELECTED STUDIES

Table A 1 summarizes the health outcomes assessed in the selected air pollution health risk assessments (AP-HRAs). All selected AP-HRAs assess mortality impacts, while only STEs, GBD as well as WHO assess both mortality and morbidity impacts. The variety of outcomes for morbidity is higher than for mortality (see Supplementary Material). Regarding mortality, most studies assess deaths and years of life lost (YLLs). Regarding morbidity, the STEs assess multiple health outcomes, while the GBD combines the effects on multiple health outcomes into one overall indicator, namely years lived with disability (YLDs). GBD and WHO additionally used disability-adjusted life years (DALYs) as a combined mortality-morbidity indicator, which condensates the meaning of both YLLs and YLDs (1). The GBD and WHO show cause-specific health impacts (including lung cancer).

Table A 1 Summary of outcomes assessed in the selected AP-HRAS

**Short name**	**Year of analysis [1]**	**Summary of outcomes[2][3]**		
		**Mortality**	**Morbidity**	**Mixed**
STE	1993, 1996, 2000, 2005, 2010	• Deaths (except analysis 2005)<br>• Lung cancer deaths (only analysis 2000)<br>• YLLs (only analysis 2000, 2005 & 2010)<br>• Working YLLs (only analysis 2000&2010)	• Asthma attacks (all years of analysis)<br>• Bronchitis cases (all years of analysis)<br>• Days of medication (only analysis 1993)<br>• Hospital admissions (only analysis 1996 & 2010)<br>• Hospital days (except analysis 1996)<br>• Invalidity cases (only analysis 1993)<br>• RADs (all years of analysis)<br>• Symptom days (only analysis 1993)<br>• Work loss days (only analysis 1993 & 2010)	
FCAH	2010	• Lung cancer deaths		
GBD	1990-2019	• Deaths<br>• YYLs	• YLDs (by cause)	• DALYs
EEA	2009, 2011-2018	• Deaths<br>• YLLs (except 2011)		
WHO	2012, 2016	• Deaths<br>• YLLs		• DALYs
CITIES	2015	• Deaths<br>• YLLs		

Abbreviations: STE = Swiss assessment for Transport Externalities. EEA = European Environment Agency. FCAH = Federal Commission for Air Hygiene. GBD = Global Burden of Disease. WHO = World Health Organization. CITIES = AP-HRA for air pollution in around 1,000 European urban areas. YLLs = Years of life lost. DALYs = Disability-adjusted life years. RADs = Restricted activity person-days. YLDs = Years lived with disability.
[1] Single assessment for each year of analysis, except for GBD, which assessed in 2019 the whole time series 1990-2019, and EEA, which included the assessment of both 2009 and 2018 in the same report from 2020.
[2] GBD and WHO health impacts are stratified by cause and include lung cancer specific results.
[3] The health outcomes assessed by STEs depend from the year of analysis.

HEALTH IMPACTS

Table A 2 shows the all-cause mortality in adults per 100,000 all-age persons attributed to exposure to PM, O_3 and NO_2 in the most recent overlapping year of analysis of CITIES, EEA and GBD (i.e. 2015). Premature deaths per 100,000 inhabitants (all ages) attributed to O_3 in 2015 were 7% to 14% of those attributed to PM (3.6 vs. 51 for EEA and 2.8 vs. 19.7 for GBD, respectively). Regarding NO_2, the estimates were 1% to 70% of those attributed to PM, according to CITIES (0.2 vs. 14.4 for high and 30.6 vs. 43.7 for low scenario, respectively) and 25% according to EEA (12.1 vs. 51). Similar proportions can be found for years of life lost (YLLs, see Supplementary Materials).

Table A 2 Annual all-cause mortality per 100,000 all-age persons attributed to PM, O_3 and NO_2 and ratio in relation to PM (O_3 and NO_2 mortality divided by PM mortality) (Switzerland 2021).

**Type of impact [1]**	**Study**	**Mortality per 100,000 persons-year**		
		PM	O_3	NO_2
**Premature deaths**	CITIES-2015-high	14.4		0.2
	CITIES-2015-low	43.7		30.6
	EEA-2015	51.0	3.6	12.1
	GBD-2015	19.7	2.8	
**YLLs**	CITIES-2015-high	177.0		2.3
	CITIES-2015-low	539.8		377.5
	EEA-2015	519.6	40.1	127.5
	GBD-2015	294.9	37.7	

Abbreviations: YLLs = Years of life lost.
[1] PM and NO_2 for adults, i.e. ages of 30 or older for EEA and GBD and 20 or older for CITIES. O_3 for all ages.

Table A 3 and Table A 4 show the absolute annual mortality and morbidity (respectively) attributed to PM. The choice of health outcomes to be assessed has not been consistent among STEs. Thus, only one out of 25 health outcomes ever assessed by STEs (4%) are available in all five STEs (1993, 1996, 2000, 2005 and 2010), six outcomes (24%) are available in four STEs and two outcomes (8%) in three STEs. Therefore, around two thirds of the health outcomes ever assessed by STEs were available only in one or two STEs (out the five STEs reviewed). Although new evidences of health effects may arise in the future and although the capacity to make assessments for past years might be limited due to lack of data, a higher consistency in the selection of outcomes in STEs (especially regarding morbidity e.g. including broadly used outcomes such as DALYs and YLDs) would be desirable to increase comparability.

Table A 3 Annual absolute mortality attributed to PM across AP-HRAs, years and counterfactual scenarios (including all outcomes, also those that are removed in a further step because of lack of comparability with a STE) (Switzerland 2021).

**Type of impact**	**Outcome disease** [1]	**Population group** [1]	**STE**	**STE**	**STE**	**STE**	**STE**	**EEA**	**EEA**	**FCAH**	**FCAH**	**GBD**	**GBD**	**WHO**	**WHO**	**CITIES**	**CITIES**
			1993	1996	2000	2005	2010	2009	2018	2010	2010	1990	2019	2012	2016	2015	2015
										Low [2]	High [2]					Low [2]	High [2]
**Premature deaths**	All causes	Adults	5,250	3,314	3,746		2,827	4,900	3,500			3,531	1,364	1,481	2,121	968	318
		Infants			23		13					17	8	0			
		Workers					335										
	Lung cancer	Adults			311					357	255	472	240	408	206		
**Working YLLs**	All causes	Adults			5,267		2,767										
		Infants					346										
**YLLs**	All causes	Adults			40,751	46,232	28,138	55,500	38,900			61,538	20,213	25,995	31,528	11,944	3,918
		Infants			1,698	1,926	753					1,523	741	33			

Abbreviations: YLLs = Years of life lost.

[1]Age ranges of the population groups differ across AP-HRAs.

[2] FCAH and CITIES, include two assessments – respectively called high and low - because they each use a lower and a higher counterfactual scenario..

Table A 4 Annual absolute morbidity attributed to PM across AP-HRAs, years and counterfactual scenarios (including all outcomes, also those that are removed in a further step because of lack of comparability with a STE) (Switzerland 2021).

Type of impact	Outcome disease	Population group [1]	STE	STE	STE	STE	STE	GBD	GBD	WHO	WHO
			1993	1996	2000	2005	2010	1990	2019	2012	2012
**Attacks**	Asthma	Adults	3,500,000								
		Children		23,637	41,073	44,477	44,943				
**Attacks (person-days)**	Asthma	Adults		62,593			107,545				
**Cases (incidence)**	Acute bronchitis	Children	77,500								
	Chronic bronchitis	Adults		4,238	999	1,081	3,078				
**Cases (prevalence)**	Acute bronchitis	Children		45,446	39,049	41,813	17,302				
	Chronic bronchitis	Adults	55,000								
**DALYs**	All causes	Adults						69,589	27,332	28,116	34,747
		Infants						1,529	745	41	43
**Hospital admissions**	CVD	All		2,979			1,138				
	RD	All		1,308			1,131				
**Hospital days**	CVD	All	14,250		9,780	9,631	10,940				
	RD	All	16,250		5,858	5,873	9,420				
**Invalidity cases**	Chronic bronchitis	Adults	25								
**Medication intake (person-days)**	Asthma	Adults	3,750,000								
**RADs**	All causes	Adults	6,250,000	2,762,682	1,773,821	1,914,797	4,746,089				
**Symptom days**	RD	All	20,000,000								
		Children	60,000								
**Work loss days**	All causes	Workers	1,065,000				1,138,140				
**YLDs**	All causes	All						8,175	7,196		

Abbreviations: DALYs = Disability-adjusted life years. CVD = Cardio-vascular diseases. RD = Respiratory diseases. RADs = Restricted activity person-days. YLDs = Years lived with disability.

[1] Age ranges of the population groups differ across AP-HRAs.

Figure A 1 shows the annual premature deaths, Figure A 2 the YLLs attributed to PM in adults and Figure A 3 these YLLs per all-age 100,000 persons in Switzerland across AP-HRAs. Of particular interest is the case of CITIES. The absolute health impacts are lower than for STE-2010 since the assessment only covers the ten largest urban areas (instead of the whole country), but when looking at population-normalized impacts the values are higher or lower depending on the scenario.

Table A 5 and Table A 6 show the values used for the above mentioned figures .

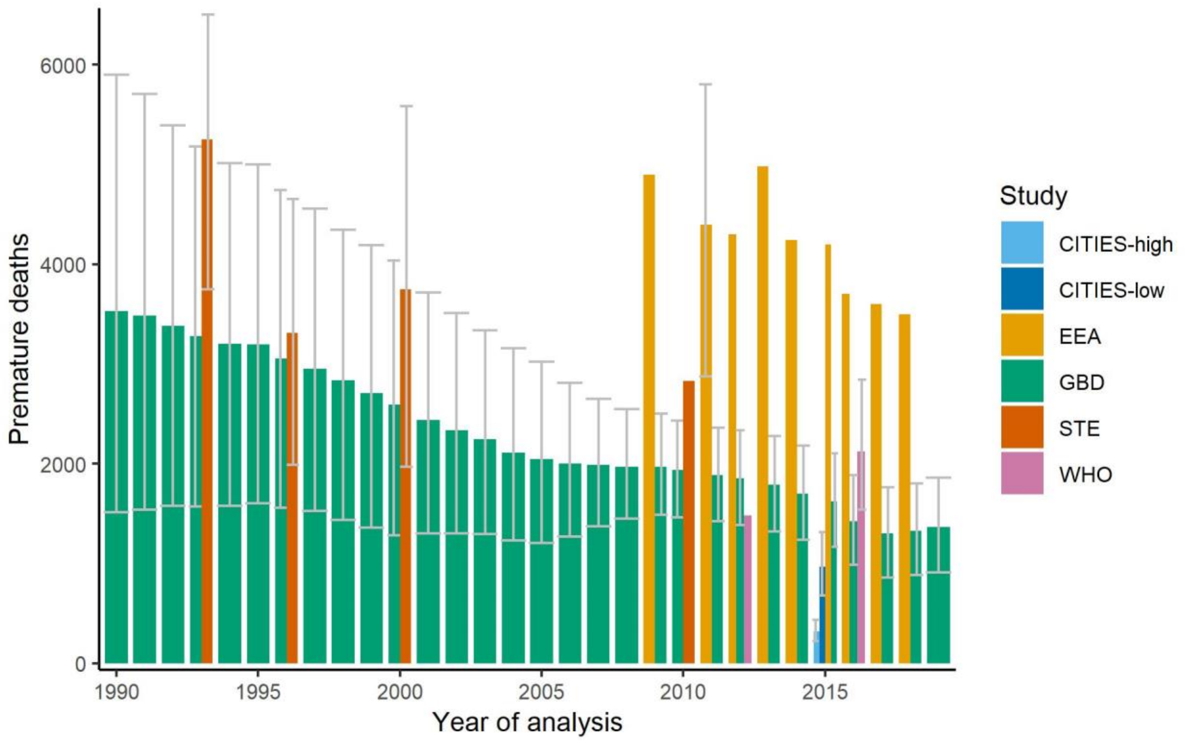

Figure A 1 Annual premature deaths in adults (≥20 years old for CITIES, ≥25 for WHO, ≥30 in the rest) attributed to PM with 95% confidence interval (if available) (Switzerland 2021).

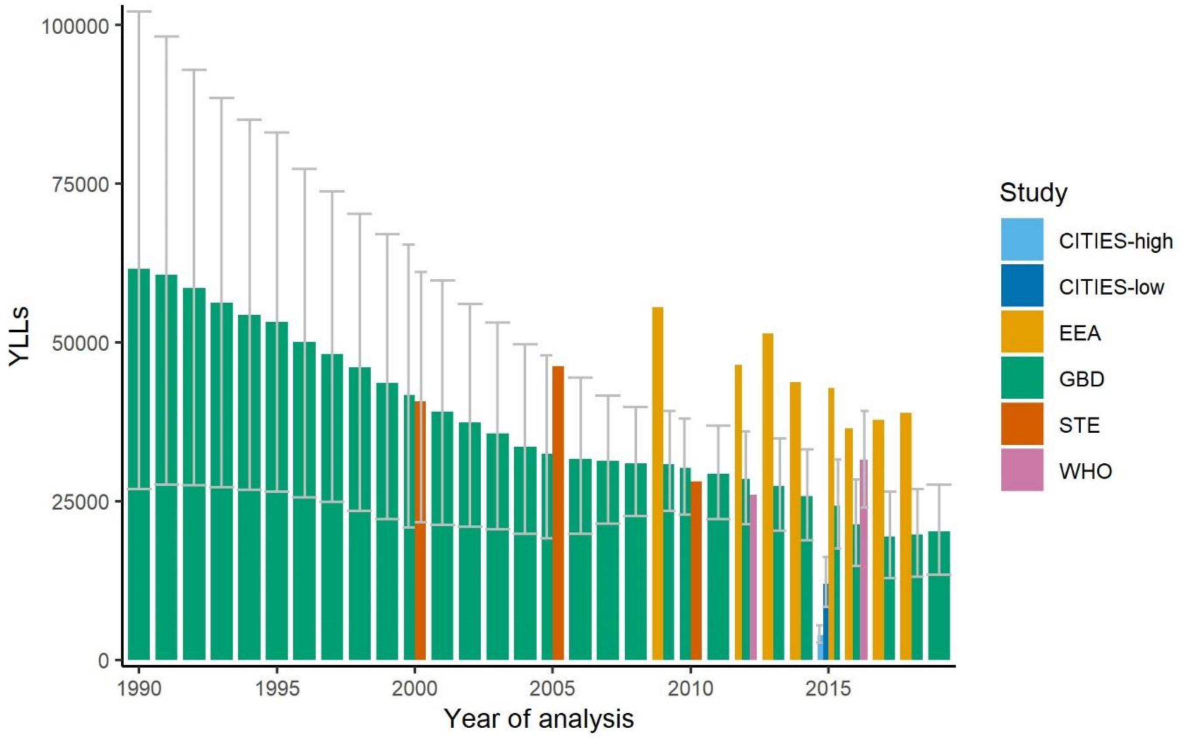

Figure A 2 Annual years of life lost due to all causes in adults (≥20 years old for CITIES, ≥25 for WHO, ≥30 in the rest) attributed to ambient PM exposure with 95% confidence interval (if available) (Switzerland 2021).

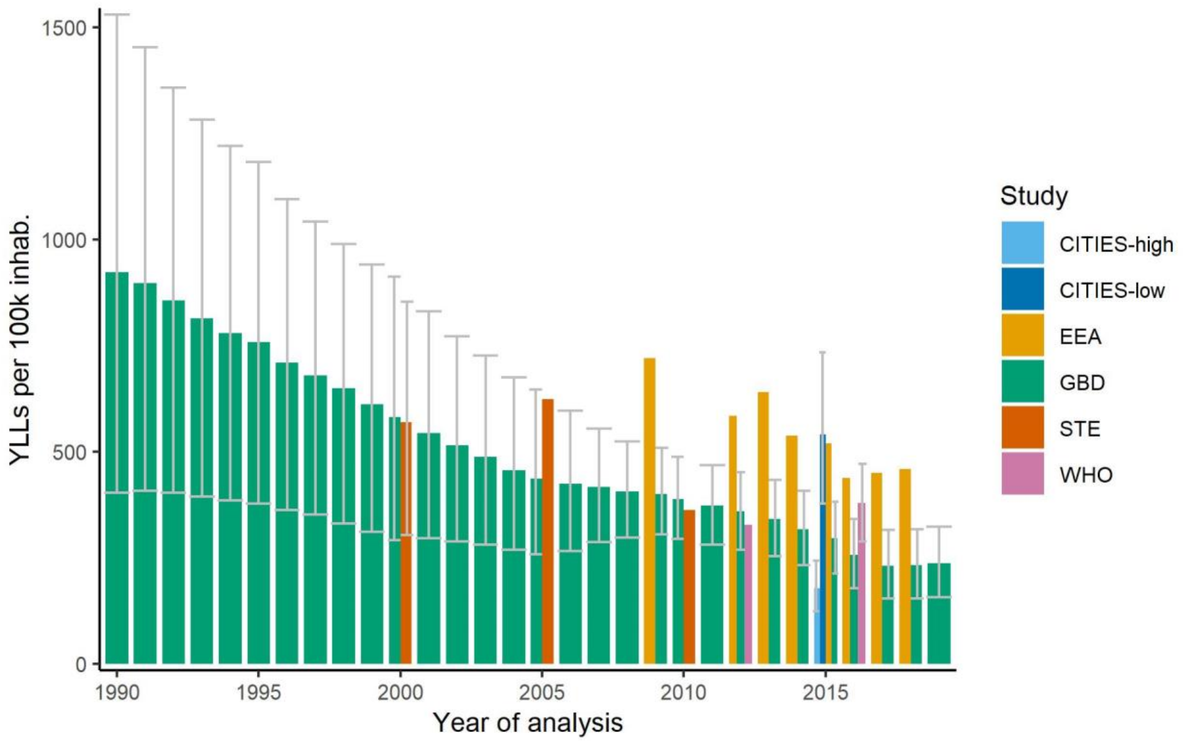

Figure A 3 Annual years of life lost per 100,000 persons in adults (≥20 years old for CITIES, ≥25 for WHO, ≥30 in the rest) attributed to PM with 95% confidence interval (if available) (Switzerland 2021).

Table A 5 Annual absolute premature deaths and per 100,000 persons attributed to PM in adults (age ≥20 for CITIES, ≥25 for WHO and ≥30 for the rest, 95% confidence interval when available) (Switzerland 2021).

Author - year of analysis	Annual deaths	Annual deaths per 100,000 persons
**CITIES-2015-high**	318 [221; 435]	14.4 [10; 19.7]
**CITIES-2015-low**	968 [677; 1316]	43.7 [30.6; 59.5]
**EEA-2009**	4900	63.6
**EEA-2011**	4394 [2876; 5803]	55.8 [36.5; 73.7]
**EEA-2012**	4300	54.1
**EEA-2013**	4980	61.9
**EEA-2014**	4240	52.1
**EEA-2015**	4200	51
**EEA-2016**	3700	44.4
**EEA-2017**	3600	42.8
**EEA-2018**	3500	41.3
**GBD-1990**	3531 [1512; 5901]	52.9 [22.7; 88.4]
**GBD-1991**	3482 [1538; 5705]	51.5 [22.8; 84.4]
**GBD-1992**	3382 [1577; 5395]	49.4 [23.1; 78.8]
**GBD-1993**	3282 [1568; 5183]	47.5 [22.7; 75]
**GBD-1994**	3200 [1580; 5015]	45.9 [22.7; 72]
**GBD-1995**	3194 [1600; 4997]	45.5 [22.8; 71.2]
**GBD-1996**	3056 [1560; 4742]	43.3 [22.1; 67.1]
**GBD-1997**	2951 [1524; 4555]	41.7 [21.5; 64.3]
**GBD-1998**	2835 [1434; 4343]	40 [20.2; 61.2]
**GBD-1999**	2708 [1358; 4192]	38 [19.1; 58.8]
**GBD-2000**	2593 [1284; 4038]	36.2 [17.9; 56.4]
**GBD-2001**	2440 [1300; 3715]	33.9 [18.1; 51.6]
**GBD-2002**	2338 [1299; 3510]	32.2 [17.9; 48.4]
**GBD-2003**	2245 [1297; 3339]	30.7 [17.7; 45.7]
**GBD-2004**	2108 [1233; 3159]	28.6 [16.7; 42.9]
**GBD-2005**	2043 [1206; 3022]	27.5 [16.3; 40.8]
**GBD-2006**	2004 [1266; 2810]	26.9 [17; 37.7]
**GBD-2007**	1988 [1373; 2648]	26.5 [18.3; 35.3]
**GBD-2008**	1972 [1450; 2549]	26 [19.1; 33.6]
**GBD-2009**	1972 [1488; 2500]	25.6 [19.3; 32.5]
**GBD-2010**	1934 [1463; 2433]	24.8 [18.8; 31.2]
**GBD-2011**	1884 [1421; 2359]	23.9 [18.1; 30]
**GBD-2012**	1851 [1384; 2337]	23.3 [17.4; 29.4]
**GBD-2013**	1791 [1322; 2277]	22.3 [16.4; 28.3]
**GBD-2014**	1699 [1237; 2180]	20.9 [15.2; 26.8]
**GBD-2015**	1624 [1164; 2102]	19.7 [14.1; 25.5]
**GBD-2016**	1426 [985; 1887]	17.1 [11.8; 22.7]
**GBD-2017**	1301 [860; 1765]	15.4 [10.2; 21]
**GBD-2018**	1328 [882; 1804]	15.6 [10.4; 21.3]
**GBD-2019**	1364 [911; 1857]	16 [10.7; 21.7]
**STE-1993**	5250 [3750; 6500]	76 [54.3; 94.1]
**STE-1996**	3314 [1986; 4651]	46.9 [28.1; 65.9]
**STE-2000**	3746 [1968; 5587]	52.3 [27.5; 78]
**STE-2010**	2827	36.3
**WHO-2012**	1481	18.6
**WHO-2016**	2121 [1541; 2843]	25.5 [18.5; 34.1]

Table A 6 Annual absolute YLLs and per 100,000 persons attributed to PM in adults (age ≥20 for CITIES, ≥25 for WHO and ≥30 for the rest, 95% confidence interval when available) (Switzerland 2021).

Author - year of analysis	YLLs	Annual YLLs per 100,000 persons
**CITIES-2015-high**	3918 [2723; 5365]	177 [123.1; 242.4]
**CITIES-2015-low**	11944 [8361; 16242]	539.8 [377.9; 734]
**EEA-2009**	55500	720.6
**EEA-2012**	46500	584.6
**EEA-2013**	51400	639.4
**EEA-2014**	43700	536.9
**EEA-2015**	42800	519.6
**EEA-2016**	36500	438.3
**EEA-2017**	37800	449
**EEA-2018**	38900	458.5
**GBD-1990**	61538 [26839; 102077]	922.1 [402.1; 1529.5]
**GBD-1991**	60630 [27548; 98151]	897.3 [407.7; 1452.5]
**GBD-1992**	58553 [27520; 92899]	855.7 [402.2; 1357.6]
**GBD-1993**	56209 [27200; 88539]	813.7 [393.8; 1281.7]
**GBD-1994**	54322 [26796; 85059]	779.5 [384.5; 1220.6]
**GBD-1995**	53191 [26474; 83035]	757.8 [377.2; 1183]
**GBD-1996**	50118 [25595; 77306]	709.6 [362.4; 1094.6]
**GBD-1997**	48153 [24913; 73824]	680 [351.8; 1042.5]
**GBD-1998**	46028 [23492; 70227]	648.6 [331; 989.6]
**GBD-1999**	43607 [22112; 67046]	612.1 [310.4; 941.2]
**GBD-2000**	41685 [20848; 65396]	581.8 [291; 912.8]
**GBD-2001**	39137 [21253; 59763]	543.7 [295.3; 830.3]
**GBD-2002**	37332 [20919; 56005]	514.5 [288.3; 771.9]
**GBD-2003**	35683 [20497; 53133]	487.9 [280.2; 726.5]
**GBD-2004**	33531 [19784; 49667]	455.3 [268.6; 674.4]
**GBD-2005**	32390 [19096; 47941]	436.8 [257.5; 646.5]
**GBD-2006**	31641 [19807; 44436]	424.2 [265.5; 595.7]
**GBD-2007**	31275 [21459; 41613]	416.5 [285.8; 554.2]
**GBD-2008**	30870 [22608; 39792]	406.5 [297.7; 524]
**GBD-2009**	30845 [23414; 39188]	400.5 [304; 508.8]
**GBD-2010**	30195 [22845; 37963]	387.8 [293.4; 487.6]
**GBD-2011**	29275 [22134; 36827]	372 [281.2; 467.9]
**GBD-2012**	28522 [21361; 35945]	358.6 [268.5; 451.9]
**GBD-2013**	27428 [20321; 34851]	341.2 [252.8; 433.5]
**GBD-2014**	25802 [18857; 33118]	317 [231.7; 406.9]
**GBD-2015**	24295 [17473; 31479]	294.9 [212.1; 382.1]
**GBD-2016**	21321 [14746; 28366]	256 [177.1; 340.7]
**GBD-2017**	19466 [12887; 26524]	231.2 [153.1; 315]
**GBD-2018**	19748 [13053; 26930]	232.8 [153.9; 317.4]
**GBD-2019**	20213 [13406; 27577]	236.6 [156.9; 322.7]
**STE-2000**	40751 [21662; 61087]	568.8 [302.4; 852.6]
**STE-2005**	46232	623.5
**STE-2010**	28138	361.4
**WHO-2012**	25995	326.8
**WHO-2016**	31528 [23956; 39220]	378.6 [287.7; 471]

Table A 7 shows the annual morbidity impacts per 100,000 all-age persons attributed to PM in Switzerland and the ratios of these values in relation to the reference value of the last available STE. Morbidity outcomes of STEs were not assessed in other AP-HRAs. The ratios STE morbidity impacts in relation to STE-2010 range from 0.35 to 2.9. The values in STE-1993 are higher than in STE-2010, while most impacts in STE-2000 and STE-2005 are lower.

Table A 7 Annual morbidity impacts attributed to PM across AP-HRAs and years expressed as per 100,000 all-age persons and as a ratio in relation to the reference value (most recent STE, in bold). The ratio is calculated by dividing the AP-HRA value by the reference value (Switzerland 2021).

Type of impact	Outcome disease	Population group [1]	STE 1993	STE 1996	STE 2000	STE 2005	STE 2010
Morbidity **per 100,000 persons**							
**Attacks**	Asthma	Adults		335	573	600	**577**
		Children		886			**1,381**
**Cases (incidence)**	Chronic bronchitis	Adults		60	14	15	**40**
	Acute bronchitis	Children		644	545	564	**222**
**Hospital admissions**	CVD	All		42			**15**
	RD	All		19			**15**
**Hospital days**	CVD	All	206		137	130	**141**
	RD	All	235		82	79	**121**
**RADs**	All causes	Adults	90,475	39,118	24,759	25,823	**60,958**
**Work loss days**	All causes	Workers	15,417				**14,618**
Ratio in relation to reference value (last STE) [2]							
**Attacks**	Asthma	Adults		0.58	0.99	1.04	**1**
		Children		0.64			**1**
**Cases (incidence)**	Chronic bronchitis	Adults		1.52	0.35	0.37	**1**
	Acute bronchitis	Children		2.90	2.45	2.54	**1**
**Hospital admissions**	CVD	All		2.89			**1**
	RD	All		1.28			**1**
**Hospital days**	CVD	All	1.47		0.97	0.92	**1**
	RD	All	1.94		0.68	0.65	**1**
**RADs**	All causes	Adults	1.48	0.64	0.41	0.42	**1**
**Work loss days**	All causes	Workers	1.05				**1**

Abbreviations: CVD = Cardio-vascular diseases. RD = Respiratory diseases. RADs = Restricted activity person-days.

[1] Age ranges of the population groups differ across AP-HRAs.

[2] Examples for interpretation of the ratio: 1.1 = 1.1 times the ref. value = 10% higher. 2.0 = 2 times the ref. value = 100% higher. 0.4 = 0.4 times the ref. value = 60% lower.

POPULATION EXPOSURE AND COUNTERFACTUAL SCENARIO

As Table A 3 shows, the population exposure has decreased over time in Switzerland. Regarding differences across specific AP-HRAS in overlapping years, the STE population exposure is higher than the GBD concentration in 2010 and 2005, while it is lower in 2000. The STE population exposure is higher to the FCAH value in 2010. WHO, EEA and CITIES have no overlapping year with STEs, but they can be compared with GBD. Thus, the population exposure for WHO is lower than for GBD in 2016 and lower for CITIES in 2015. EEA values are rather similar to GBD values, being higher or lower depending on the year. WHO-2012 did not publish the annual population-weighted mean used in the assessment for Switzerland, but only the median for a different year. Therefore, WHO-2012 has not been included in the comparison of population exposures.

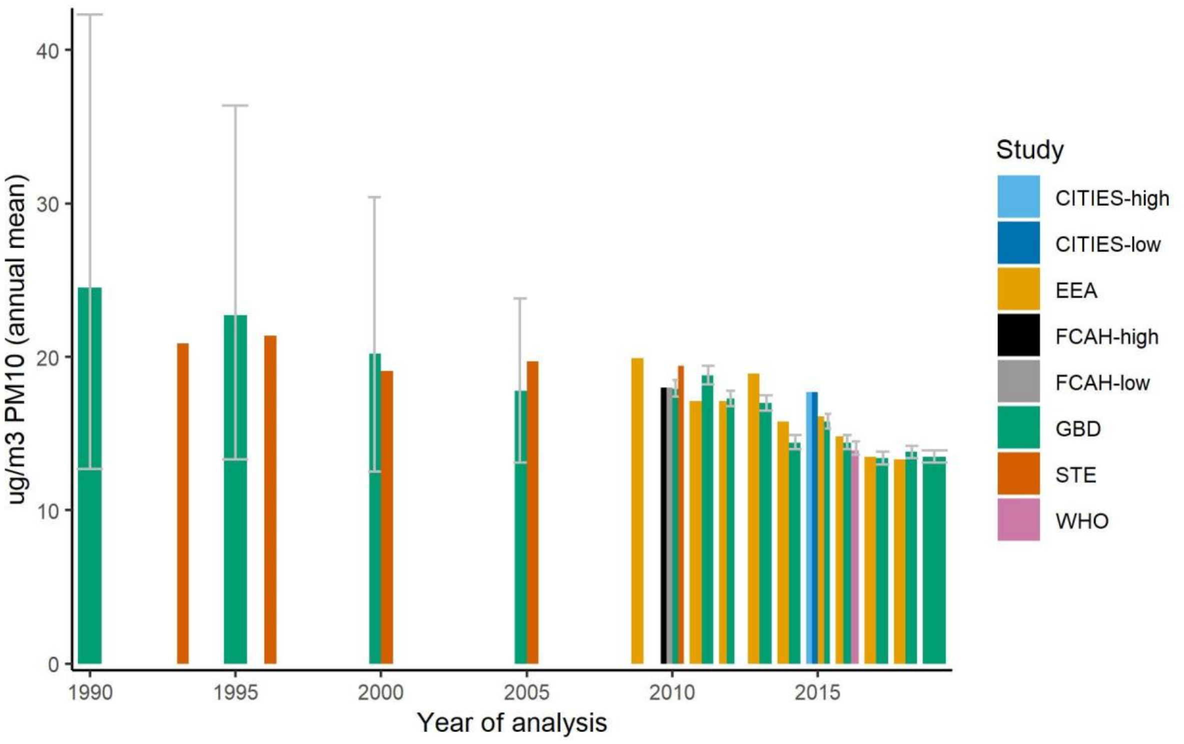

Figure A 4 Annual population-weighted mean PM_{10} concentration over time in the selected studies with 95% confidence interval (if available) (Switzerland 2021).

Table A 8, Table A 9, Table A 10 and

Table A 11 show the population exposure and counterfactual scenario for PM_{10}, $PM_{2.5}$, O_3, NO_2, respectively.

As metric for O_3 concentrations, the GBD uses the daily 8-hour maximum (MDA8) in parts per billion (ppb) during the warm season, defined as the six months with the highest average O_3 levels (2), while the EEA reports use the yearly accumulated MDA8 in μg/m^3 exceeding 35 ppb (SOMO35). Since 1 ppb is equivalent to 2.00 μg/m^3 in the case of O_3 , the 35 ppb are equal to 70 μg/m^3 (3).

The difference between population exposure and counterfactual scenarios of NO_2 in EEA assessments for 2016, 2017 and 2018 are negative. Therefore, we did not use these values for normalizing absolute health impacts. EEA aggregated effects of grids inside countries (4). Thus, the negative value of the exposure difference implies that population exposure is lower or higher than the counterfactual scenario depending on the grid.

Table A 8 PM_{10} concentrations (population-weighted annual mean) in Switzerland (Switzerland 2021).

Author-year of analysis	Age group	Concentration (µg/m³ PM_{10})		
		Population exposure	Counterfactual scenario	Difference
**FCAH-2010-high**	All	18	7.5	10.5
**FCAH-2010-low**	All	18	3.3	14.7
**STE-1993**	All	20.9		
**STE-1996**	All	21.4	7.5	13.9
**STE-2000**	<15	18.7	7.5	11.2
**STE-2000**	≥30	19.2	7.5	11.7
**STE-2000**	All	19.1	7.5	11.6
**STE-2005**	<15	19.2	7.5	11.7
**STE-2005**	≥30	19.8	7.5	12.3
**STE-2005**	All	19.7	7.5	12.2
**STE-2010**	<15	19.4	7.5	11.9
**STE-2010**	≥30	19.5	7.5	12
**STE-2010**	All	19.4	7.5	11.9

Table A 9 $PM_{2.5}$ concentration (population-weighted annual mean, all ages) in Switzerland (Switzerland 2021).

Author-year of analysis	Original concentration (µg/m³ $PM_{2.5}$)		Re-scaled concentration (µg/m³ PM_{10})		
	Population exposure [1]	Counter-factual scenario [1]	Population exposure	Counter-factual scenario	Difference
**CITIES-2015-high**	13	10	17.7	13.6	4.1
**CITIES-2015-low**	13	3.7	17.7	5	12.7
**EEA-2009**	14.6	0	19.9	0	19.9
**EEA-2011**	12.6	0	17.1	0	17.1
**EEA-2012**	12.6	0	17.1	0	17.1
**EEA-2013**	13.9	0	18.9	0	18.9
**EEA-2014**	11.6	0	15.8	0	15.8
**EEA-2015**	11.8	0	16.1	0	16.1
**EEA-2016**	10.9	0	14.8	0	14.8
**EEA-2016**	9.9	0	13.5	0	13.5
**EEA-2018**	9.8	0	13.3	0	13.3
**GBD-1990**	18 [9.3; 31.1]	4.2 [2.4; 5.9]	24.5 [12.7; 42.3]	5.6 [3.3; 8]	18.8 [9.4; 34.2]
**GBD-1995**	16.7 [9.8; 26.7]	4.2 [2.4; 5.9]	22.7 [13.3; 36.4]	5.6 [3.3; 8]	17.1 [10; 28.3]
**GBD-2000**	14.9 [9.2; 22.3]	4.2 [2.4; 5.9]	20.2 [12.5; 30.4]	5.6 [3.3; 8]	14.6 [9.3; 22.4]
**GBD-2005**	13.1 [9.6; 17.5]	4.2 [2.4; 5.9]	17.8 [13.1; 23.8]	5.6 [3.3; 8]	12.1 [9.8; 15.7]
**GBD-2010**	13.2 [12.8; 13.6]	4.2 [2.4; 5.9]	17.9 [17.4; 18.5]	5.6 [3.3; 8]	12.2 [14.1; 10.4]
**GBD-2011**	13.8 [13.4; 14.3]	4.2 [2.4; 5.9]	18.8 [18.2; 19.4]	5.6 [3.3; 8]	13.2 [15; 11.4]
**GBD-2012**	12.7 [12.3; 13.1]	4.2 [2.4; 5.9]	17.3 [16.8; 17.8]	5.6 [3.3; 8]	11.6 [13.5; 9.8]
**GBD-2013**	12.5 [12.1; 12.9]	4.2 [2.4; 5.9]	17 [16.5; 17.5]	5.6 [3.3; 8]	11.3 [13.2; 9.5]
**GBD-2014**	10.6 [10.3; 10.9]	4.2 [2.4; 5.9]	14.4 [14; 14.9]	5.6 [3.3; 8]	8.8 [10.8; 6.8]
**GBD-2015**	11.6 [11.2; 11.9]	4.2 [2.4; 5.9]	15.8 [15.3; 16.3]	5.6 [3.3; 8]	10.1 [12; 8.2]
**GBD-2016**	10.6 [10.3; 10.9]	4.2 [2.4; 5.9]	14.4 [14; 14.9]	5.6 [3.3; 8]	8.8 [10.7; 6.8]
**GBD-2017**	9.9 [9.6; 10.2]	4.2 [2.4; 5.9]	13.4 [13; 13.8]	5.6 [3.3; 8]	7.8 [9.7; 5.8]
**GBD-2018**	10.2 [9.9; 10.5]	4.2 [2.4; 5.9]	13.8 [13.4; 14.2]	5.6 [3.3; 8]	8.2 [10.1; 6.2]
**GBD-2019**	9.9 [9.6; 10.2]	4.2 [2.4; 5.9]	13.5 [13.1; 13.9]	5.6 [3.3; 8]	7.8 [9.8; 5.9]
**WHO-2012**		7.3 [5.9; 8.7]		9.9 [8; 11.8]	
**WHO-2016**	10.2 [10; 10.6]	4.2 [2.4; 5.9]	13.9 [13.6; 14.5]	5.6 [3.3; 8]	8.2 [10.3; 6.4]

[1] Average of the uniform distribution with lower and upper limits of 5.9 and 8.7 µg/m3 PM2.5 for WHO-2012 and between 2.4 and 5.9 for GBD and WHO-2016.

Table A 10 Population-weighted O_3 concentrations in Switzerland (all ages) (Switzerland 2021).

**Author-year of analysis**	**Concentration**		
	**Population exposure**	**Counterfactual scenario**	**Metric & unit**
**EEA-2009**	5119		SOMO35 (µg/m^3*day)
**EEA-2011**	5435		SOMO35 (µg/m^3*day)
**EEA-2012**	4990		SOMO35 (µg/m^3*day)
**EEA-2013**	4919		SOMO35 (µg/m^3*day)
**EEA-2014**	4417		SOMO35 (µg/m^3*day)
**EEA-2015**	6170		SOMO35 (µg/m^3*day)
**EEA-2016**	4842		SOMO35 (µg/m^3*day)
**EEA-2017**	5281		SOMO35 (µg/m^3*day)
**EEA-2018**	7214		SOMO35 (µg/m^3*day)
**GBD-1990**	49.2 [48.5; 49.9]	32.4 [29.1; 35.7]	DMA8 (ppb)
**GBD-1991**	48 [47.2; 48.8]	32.4 [29.1; 35.7]	DMA8 (ppb)
**GBD-1992**	46.9 [46.2; 47.6]	32.4 [29.1; 35.7]	DMA8 (ppb)
**GBD-1993**	45.5 [44.8; 46.1]	32.4 [29.1; 35.7]	DMA8 (ppb)
**GBD-1994**	46.3 [45.5; 46.9]	32.4 [29.1; 35.7]	DMA8 (ppb)
**GBD-1995**	47.5 [46.8; 48.1]	32.4 [29.1; 35.7]	DMA8 (ppb)
**GBD-1996**	47.9 [47.2; 48.6]	32.4 [29.1; 35.7]	DMA8 (ppb)
**GBD-1997**	48.7 [48; 49.4]	32.4 [29.1; 35.7]	DMA8 (ppb)
**GBD-1998**	48.5 [47.8; 49.2]	32.4 [29.1; 35.7]	DMA8 (ppb)
**GBD-1999**	48.3 [47.6; 48.9]	32.4 [29.1; 35.7]	DMA8 (ppb)
**GBD-2000**	47.3 [46.6; 48]	32.4 [29.1; 35.7]	DMA8 (ppb)
**GBD-2001**	47.3 [46.6; 48]	32.4 [29.1; 35.7]	DMA8 (ppb)
**GBD-2002**	50.8 [50.1; 51.5]	32.4 [29.1; 35.7]	DMA8 (ppb)
**GBD-2003**	51.2 [50.5; 51.9]	32.4 [29.1; 35.7]	DMA8 (ppb)
**GBD-2004**	51.4 [50.7; 52.1]	32.4 [29.1; 35.7]	DMA8 (ppb)
**GBD-2005**	49 [48.3; 49.7]	32.4 [29.1; 35.7]	DMA8 (ppb)
**GBD-2006**	48.2 [47.5; 48.9]	32.4 [29.1; 35.7]	DMA8 (ppb)
**GBD-2007**	47.6 [47; 48.3]	32.4 [29.1; 35.7]	DMA8 (ppb)
**GBD-2008**	46.8 [46; 47.4]	32.4 [29.1; 35.7]	DMA8 (ppb)
**GBD-2009**	47.1 [46.3; 47.8]	32.4 [29.1; 35.7]	DMA8 (ppb)
**GBD-2010**	47.7 [46.9; 48.4]	32.4 [29.1; 35.7]	DMA8 (ppb)
**GBD-2011**	47.4 [46.7; 48.1]	32.4 [29.1; 35.7]	DMA8 (ppb)
**GBD-2012**	48 [47.3; 48.8]	32.4 [29.1; 35.7]	DMA8 (ppb)
**GBD-2013**	48.2 [47.4; 49]	32.4 [29.1; 35.7]	DMA8 (ppb)
**GBD-2014**	49.4 [48.6; 50.1]	32.4 [29.1; 35.7]	DMA8 (ppb)
**GBD-2015**	48.3 [47.5; 49.1]	32.4 [29.1; 35.7]	DMA8 (ppb)
**GBD-2016**	47.8 [47.1; 48.6]	32.4 [29.1; 35.7]	DMA8 (ppb)
**GBD-2017**	46.7 [46.2; 47.2]	32.4 [29.1; 35.7]	DMA8 (ppb)
**GBD-2018**	48.3 [47.5; 49]	32.4 [29.1; 35.7]	DMA8 (ppb)
**GBD-2019**	48.3 [47.5; 49.1]	32.4 [29.1; 35.7]	DMA8 (ppb)

Table A 11 NO_2 concentration in Switzerland (population-weighted annual mean, all-ages) (Switzerland 2021).

Author-year of analysis	Population exposure (µg/m³)	Counterfactual scenario (µg/m³)
**CITIES-2015-high**	27.3	40
**CITIES-2015-low**	27.3	3.5
**EEA-2009**	23.1	20
**EEA-2012**	21.6	20
**EEA-2013**	22.4	20
**EEA-2014**	20.9	20
**EEA-2015**	21.4	20
**EEA-2016**	19.7	20
**EEA-2017**	18.8	20
**EEA-2018**	17.6	20

Figure A 5 and Figure A 6 show the annual number of premature deaths and YLLs, respectively, in adults attributed to PM normalized by both population and difference between population exposure and counterfactual scenario. Differences of these double-normalized mortality impacts across AP-HRAs (and over time) become smaller than of absolute values or single normalized values by population. It implies that the difference between population exposure and counterfactual scenario largely explains the heterogeneity of health impacts.

Looking at the specific comparisons across AP-HRAs, the double-normalized mortality impacts are higher for STE than for GBD, although the difference is smaller for YLLs than for premature deaths. There are no overlapping years for STEs and other AP-HRAs such as EEA, WHO or CITIES. Nevertheless, in close years to STE-2010, WHO and CITIES values seem to be higher in terms of YLLs and similar in terms of premature deaths. Both impacts are rather similar when comparing STE-2010 with EEA, while EEA values are rather similar. Double normalized premature deaths are similar across these AP-HRAs. Anyway, the confidence intervals (when available) are rather wide. Therefore, differences across AP-HRAs might not be statistically different.

We normalized the health impacts per 100,000 (all-age) persons by dividing by 10 units of difference in concentration between population exposure and counterfactual scenario, assuming a linear relationship. We used concentration data from the selected AP-HRAs. Some of these concentration data were expressed with CI. However, the CI of the normalized health impacts (when available) only refer to the lower and upper bound of the impacts and not to the concentration data (we only use mean values of concentration data for normalizing.

Table A 12 and Table A 13 show the values used for the estimation of premature normality normalized per population and PM concentration. Table A 14 and Table A 15 show all health outcomes across normalized by population and PM concentration.

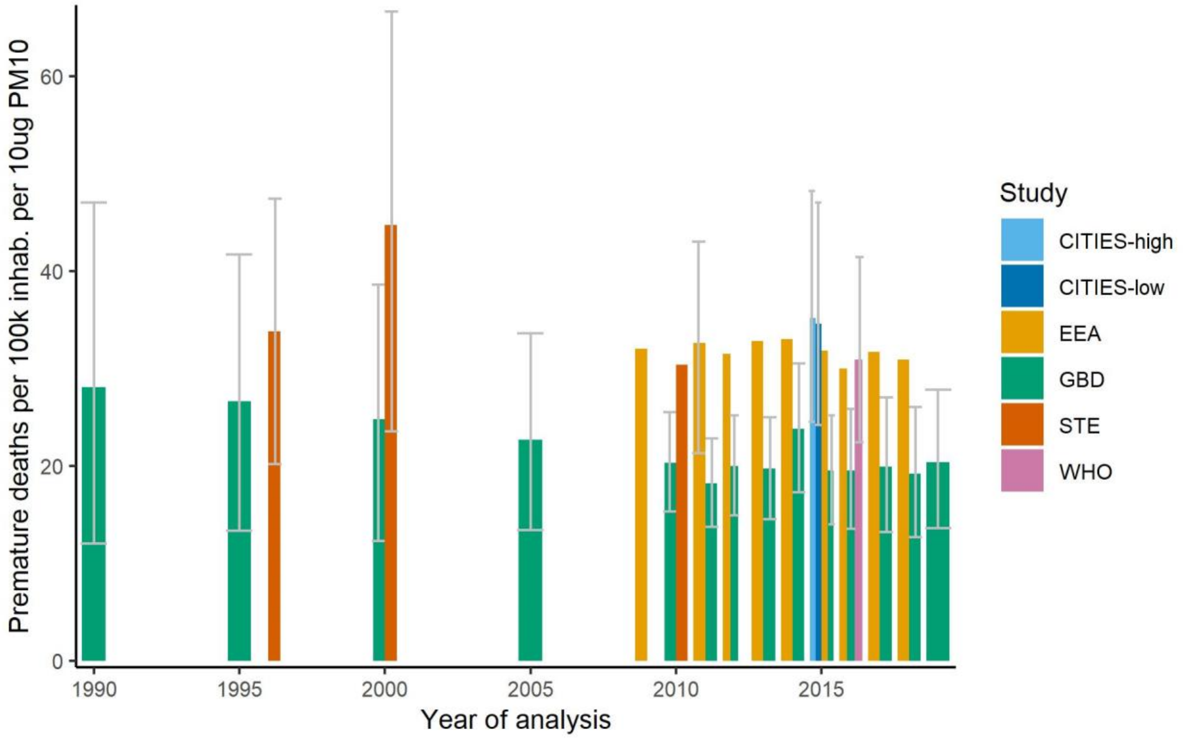

Figure A 5 Annual premature deaths per 100,000 persons and per 10 μg/m³ PM_{10} in adults (≥20 years old for CITIES, ≥25 for WHO, ≥30 in the rest) attributed to PM with 95% confidence interval (if available) (Switzerland 2021).

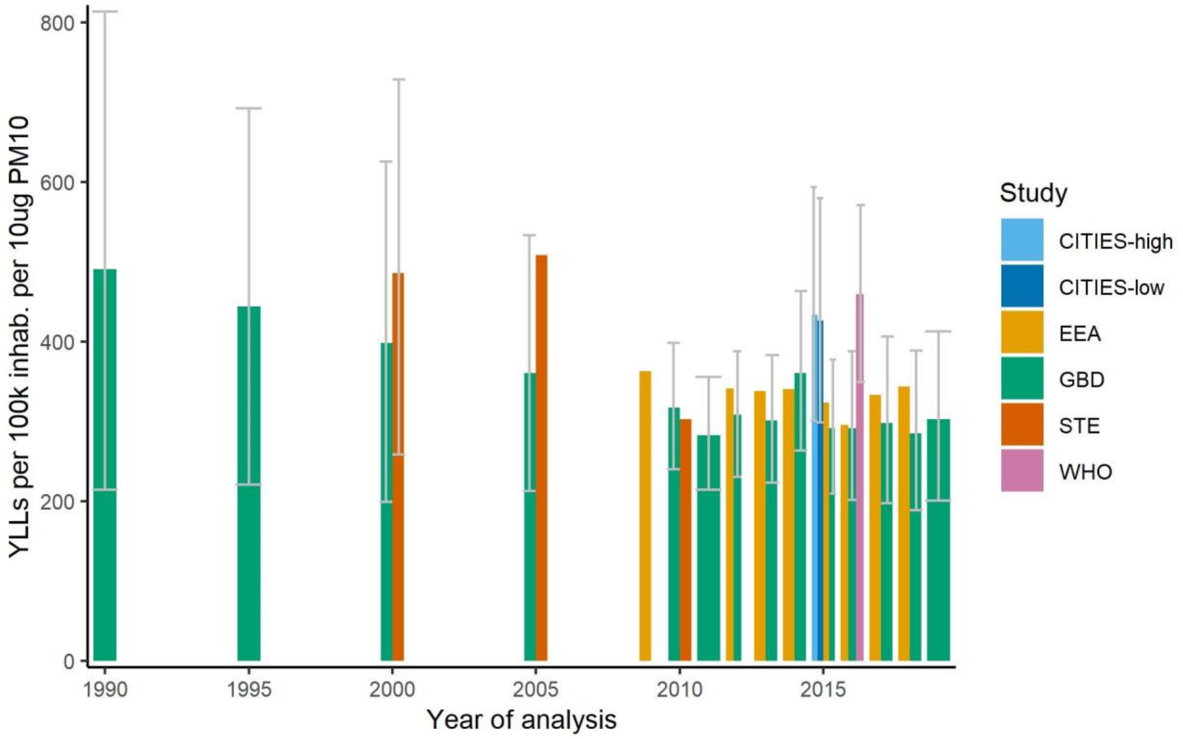

Figure A 6 Annual years of life lost per 100,000 persons and per 10 μg/m³ PM_{10} in adults (≥20 years old for CITIES, ≥25 for WHO, ≥30 in the rest) attributed to PM with 95% confidence interval (if available) (Switzerland 2021).

Table A 12 Annual premature deaths per 100,000 persons and 10 μg/m^3 PM_{10} attributed to PM in adults (age ≥20 for CITIES, ≥25 for WHO and ≥30 for the rest, 95% confidence interval when available) (Switzerland 2021).

Author - year of analysis	Annual deaths per 100,000 persons and 10 μg/m^3 PM_{10}
**CITIES-2015-high**	35.2 [24.5; 48.2]
**CITIES-2015-low**	34.6 [24.2; 47]
**EEA-2009**	32
**EEA-2012**	32.6 [21.3; 43]
**EEA-2013**	31.5
**EEA-2014**	32.8
**EEA-2015**	33
**EEA-2016**	31.8
**EEA-2017**	30
**EEA-2018**	31.7
**GBD-1990**	30.9
**GBD-1991**	28.1 [12; 47]
**GBD-1992**	
**GBD-1993**	
**GBD-1994**	
**GBD-1995**	
**GBD-1996**	26.6 [13.3; 41.7]
**GBD-1997**	
**GBD-1998**	
**GBD-1999**	
**GBD-2000**	
**GBD-2001**	24.8 [12.3; 38.6]
**GBD-2002**	
**GBD-2003**	
**GBD-2004**	
**GBD-2005**	
**GBD-2006**	22.7 [13.4; 33.6]
**GBD-2007**	
**GBD-2008**	
**GBD-2009**	
**GBD-2010**	
**GBD-2011**	20.3 [15.3; 25.5]
**GBD-2012**	18.2 [13.7; 22.8]
**GBD-2013**	20 [14.9; 25.2]
**GBD-2014**	19.7 [14.5; 25]
**GBD-2015**	23.8 [17.3; 30.5]
**GBD-2016**	19.5 [14; 25.2]
**GBD-2017**	19.5 [13.5; 25.8]
**GBD-2018**	19.9 [13.2; 27]
**GBD-2019**	19.2 [12.7; 26]
**STE-2000**	20.4 [13.6; 27.8]
**STE-2005**	47.8 [34.2; 59.2]
**STE-2010**	33.8 [20.2; 47.4]
**WHO-2016**	30.9 [22.4; 41.4]

Table A 13 Annual YLLs per 100,000 persons and 10 µg/m^3 PM_{10} attributed to PM in adults (age ≥20 for CITIES, ≥25 for WHO and ≥30 for the rest, 95% confidence interval when available) (Switzerland 2021).

Author - year of analysis	Annual YLLs per 100,000 persons and 10 µg/m^3 PM_{10}
**CITIES-2015-high**	433.7 [301.5; 593.9]
**CITIES-2015-low**	426.6 [298.6; 580.1]
**EEA-2009**	362.8
**EEA-2012**	341
**EEA-2013**	338.1
**EEA-2014**	340.2
**EEA-2015**	323.6
**EEA-2016**	295.6
**EEA-2017**	333.3
**EEA-2018**	343.9
**GBD-1990**	490.3 [213.8; 813.2]
**GBD-1991**	
**GBD-1992**	
**GBD-1993**	
**GBD-1994**	
**GBD-1995**	443.6 [220.8; 692.4]
**GBD-1996**	
**GBD-1997**	
**GBD-1998**	
**GBD-1999**	
**GBD-2000**	398.5 [199.3; 625.2]
**GBD-2001**	
**GBD-2002**	
**GBD-2003**	
**GBD-2004**	
**GBD-2005**	360.1 [212.3; 533]
**GBD-2006**	
**GBD-2007**	
**GBD-2008**	
**GBD-2009**	
**GBD-2010**	316.7 [239.6; 398.1]
**GBD-2011**	282.8 [213.8; 355.7]
**GBD-2012**	307.8 [230.5; 387.9]
**GBD-2013**	301.3 [223.3; 382.9]
**GBD-2014**	360.7 [263.6; 463]
**GBD-2015**	291.3 [209.5; 377.5]
**GBD-2016**	291.6 [201.7; 388]
**GBD-2017**	297.9 [197.2; 405.9]
**GBD-2018**	285.1 [188.5; 388.8]
**GBD-2019**	302.2 [200.5; 412.4]
**STE-2000**	485.7 [258.2; 728.1]
**STE-2005**	508.3
**STE-2010**	302.3
**WHO-2016**	459.2 [348.9; 571.3]

Table A 14 Annual mortality per 100,000 persons and per 10 µg/m³ PM_{10} (difference between population exposure and counterfactual scenario) attributed to PM across AP-HRAs, years and counterfactual scenarios (Switzerland 2021).

**Type of impact**	**Outcome disease**	**Population group [1]**	**STE**	**STE**	**STE**	**STE**	**EEA**	**EEA**	**FCAH**	**FCAH**	**GBD**	**GBD**	**WHO**	**CITIES**	**CITIES**
			1996	2000	2005	2010	2009	2018	2010	2010	1990	2019	2016	2015	2015
									Low [2]	High [2]				Low [2]	High [2]
**Premature deaths**	All causes	Adults	34	45		30	32	31			28	20	31	35	35
		Infants		0.3		0.1					0.1	0.1			
		Workers				4									
	Lung cancer	Adults		4					3	3	4	4	3		
**Working YLLs**	All causes	Adults		63		30									
		Infants				4									
**YLLs**	All causes	Adults		486	508	302	363	344			490	302	459	427	434
		Infants		21	22	8					12	11	31		

Abbreviations: YLLs = Years of life lost.

[1] Age ranges of the population groups differ across AP-HRAs.

[2] FCAH and CITIES, include two assessments – respectively called high and low - because they each use a lower and a higher counterfactual scenario.

Table A 15 Annual morbidity per 100,000 persons and per 10 µg/m³ PM_{10} (difference between population exposure and counterfactual scenario) attributed to exposure to PM across AP-HRAs, years and counterfactual scenarios (Switzerland 2021).

**Type of impact**	**Outcome disease**	**Population group** [1]	**STE**	**STE**	**STE**	**STE**	**GBD**	**GBD**	**WHO**
			1996	2000	2005	2010	1990	2019	2016
**Attacks**	Asthma	Adults							
		Children	241	490	489	483			
**Attacks (person-days)**	Asthma	Adults	638			1,163			
**Cases (incidence)**	Acute bronchitis	Children							
	Chronic bronchitis	Adults	43	12	12	33			
**Cases (prevalence)**	Acute bronchitis	Children	463	487	480	187			
	Chronic bronchitis	Adults							
**DALYs**	All causes	Adults					554	409	506
		Infants					12	11	1
**Hospital admissions**	CVD	All	30			12			
	RD	All	13			12			
**Hospital days**	CVD	All		118	107	118			
	RD	All		70	65	101			
**Invalidity cases**	Chronic bronchitis	Adults							
**Medication intake (person-days)**	Asthma	Adults							
**RADs**	All causes	Adults	28,143	21,143	21,054	50,986			
**Symptom days**	RD	All							
		Children							
**Work loss days**	All causes	Workers				12,227			
**YLDs**	All causes	All					65	108	

Note: DALYs = Disability-adjusted life years. CVD = Cardio-vascular diseases. RD = Respiratory diseases. RADs = Restricted activity days YLDs = Years lived with disability.
[1] Age ranges of the population groups differ across AP-HRAs.

CONCENTRATION-RESPONSE FUNCTION

Table A 17 shows the CRFs in form of relative risk of both mortality and morbidity impacts attributed to outdoor PM_{10} exposure, including the lower and upper bound of the CI and re-scaled from $PM_{2.5}$ to PM_{10} (if needed).

Table A 18 shows the excess relative risk (relative risk minus one) and the corresponding ratios for morbidity outcomes. The prevalence of bronchitis in children and the restricted activity person-days (RADs) show the largest differences between STE-2000 and STE-2010 (being the STE-2010 in both cases lower). For bronchitis in children, STE-2000 carried out an own meta-analysis based on six studies (5-10), while STE-2010 based on a more recent study in nine countries (11). For RAD, STE-2010 used a different definition and an estimate from an older study than the one used in STE-2000, following WHO recommendations.

Table A 16 summarizes the main specific methodological differences among AP-HRAs.

Table A 16 Main differences in methodological approaches for the quantification of health impacts in the AP-HRAs (Switzerland 2021).

Particularities		STE	STE	STE	EEA	GBD	WHO	CITIES
**Topic**	**Description**	1996	2000	2010	2009 - 2018	1990 - 2019	2012 & 2016	2015
**All-cause CRF**	Aggregation of disease-specific for all-cause mortality					X	X	
	Aggregation of stratified sex-and/or sex specific mortality			X	X	X	X	X
**Shape of exposure-response function**	Linear exposure-response function (instead of log-linear)	X	X					
	Integrated exposure-response function for CRF					X	X	
**PAF**	Different concentrations across spatial units of analysis (instead of single exposure level)					X	X	X
**Quantification of mortality impacts**	Life table approach for premature deaths			X				
	Life table approach for YLLs			X	X			X
	Life table approach for YLLs with discount rate			X				

Notes: We excluded STE-1993, STE-2005 and FCAH from this table due to the following reasons. No information on these specific aspects of the methodology is available for STE-1993. We assumed that STE-2005 uses the same methodology as STE-2000, because STE-2005 is an update of some input data, which mainly replicate the methodology of the STE-2000. FCAH focuses on lung cancer using a very simplified method (see Supplementary Material).

Health impacts can be calculated as in Equation A 1, by multiplying the reported baseline health data and the population attributable fraction (PAF). The health data are equal to baseline health rates (per inhabitant) multiplied by the population at risk at the corresponding age Equation A 2. The PAF can be calculated for the whole population, when considering only an average concentration as in as in STE-2010 (only for morbidity), STE-2000 and EEA (Equation A 3). Alternatively, when considering multiple exposure levels in grids (Equation A 4 and Equation A 5), PAF can be calculated using the Miettinen's formula (12) and the Levin's formula (13), but both are mathematically equivalent (14). GBD, CITIES an WHO use this PAF for multiple

exposure levels (GBD and CITIES based on Miettinen's and WHO based on Levin's formulation). It should be noted that PAFs for counterfactual cases different to zero can also be referred as Potential Impact Factor (PIF) (15).

The exposure-response functions (CRF) enables the estimation of relative risk values for concentrations different to the one provided in the literature (e.g. normally 10 µg/m^3 PM). These functions can vary depending on the study. Thus, STE-2000 (ARE, BAG et al. 2004) apply Equation A 6, while STE-2010, EEA, and CITIES use Equation A 7. In contrast, GBD and WHO applies an integrated exposure response function to derive the relative risk (Equation A 8). FCAH uses a very simplified method based on excess rates as in Equation A 9.

STE 2010 applies a life table approach for assessing premature deaths and years of life lost considering separately adult males, adult females and infants. STE-2000 and consequently the short update for 2005 as well as EEA and CITIES use this life table approach for assessing years of life lost. This life table approach involve more numerous and more complex calculations as the general approach presented in the equations above. Therefore, such equations are not normally published, being EEA an exception.

Equation A 1 Health impact.

$$I = H_B * PAF_E$$

I = Assessed health impact of an air pollution exposure E.
H_B = Baseline health data (e.g. reported annual hospital days due to respiratory diseases in Switzerland) including the effect of exposure.
AF_E= Attributable fraction for an exposure E. By definition: 1- H_A/H_B, being H_A the initial baseline health data without the effect of exposure E.

Equation A 2 Baseline health data.

$$H_B = HR_B * PR$$

H_B = Baseline health rate.
HR_B = Baseline health rate, normalized by population (at risk).
PR = Population (at risk), i.e. population within a specific age range determined by the denominator of the baseline health rate.

Equation A 3 Population attributable fraction for single concentration level as in STE-2010 (for morbidity) (16), STE-2000 (17) and EEA (18).

$$PAF_E = 1 - \frac{1}{RR_E}$$

PAF_E = Population attributable fraction for an exposure E.
RR_E = Relative risk for an exposure E.

Equation A 4 Population attributable fraction for multiple concentration levels based on Miettinen's formula as in GBD (19) and CITIES (20).

$$PAF_E = \frac{\sum P_{B,i} * RR_i - \sum P_{A,i} * RR_i}{\sum P_{B,i} * RR_i}$$

PAF_E = Population attributable fraction for an exposure E.
$P_{B,i}$ = Proportion of the population for a population exposure i.
$P_{A,i}$ = Proportion of the population in the counterfactual case at concentration level i (normally equals to 1 for outdoor air pollution, i.e. 100% of people are exposed to counterfactual scenario) .
RR_i = Relative risk in concentration level i.

Equation A 5 Population attributable fraction for multiple concentration levels based on Levin's formula as in WHO (21).

$$PAF_E = \frac{\sum P_i * (RR_i - 1)}{1 + \sum P_i * (RR_i - 1)}$$

PAF_E = Population attributable fraction for an exposure E.
P_i = Proportion of the population at concentration level i.
RR_i = Relative risk in concentration level i.

Equation A 6 Concentration-response function as in STE-2000 (17).

$$RR_E = 1 + \frac{(RR_D - 1)}{D} * (C_B - C_A)$$

RR_E = Relative risk for an exposure E.
RR_D = Relative risk for a difference in concentration D as in the literature.
D = Difference in concentration of the relative risk as in the literature (e.g. 10 in μg/m^3 for PM).
C_B = Population-weighted concentration of the pollutant (population exposure).
C_A = Minimum considered concentration (counterfactual scenario).

Equation A 7 Concentration-response function as in STE-2010 (22), EEA (23), and CITIES (20).

$$RR_E = e^{\frac{\ln(RR_D)}{D} * (C_B - C_A)}$$

RR_D = Relative risk for a concentration D as in literature.
D = Difference in concentration of the relative risk as in the literature (e.g. 10 in μg/m^3 for PM).
C_B = Population-weighted concentration of the pollutant (population exposure).
C_A = Minimum considered concentration (counterfactual scenario).

Equation A 8 Concentration-response function based in integrated exposure-response function as in GBD (19) and WHO (21).

$$RR_E = 1 + x * \left(1 - e^{y*(C_B - C_A)^z}\right)$$

C_B = Population-weighted concentration of the pollutant (population exposure).
C_A = Minimum considered concentration (counterfactual scenario).
x, y , z = Parameters estimates of the integrated exposure-response function.

Equation A 9 Health impacts as in FCAH (24).

$$I = PR * HR_B * ln(RR_D) * \frac{(C_B - C_A)}{D}$$

I = Assessed health impact of an air pollution exposure E.
HR_B = Baseline health rate (i.e. by population)
PR = Population at risk (population within a specific age range determined by the denominator of the baseline health rate).
RR_D = Relative risk for a difference in concentration D as in the literature.
D = Difference in concentration of the relative risk as in the literature (e.g. 10 in µg/m^3 for PM).
C_B = Population-weighted concentration of the pollutant (population exposure).
C_A = Minimum considered concentration (counterfactual scenario).

Table A 17 Relative risk coefficients per 10 μg/m³ PM_{10} (including lower and upper bound between squared brackets) across AP-HRAs, years (Switzerland 2021).

Type of impact	Outcome disease	Population group [1]	STE	STE	STE	STE	EEA	FCAH	GBD	CITIES
			1993	1996	2000 & 2005	2010	2009 - 2018	2010	1990 - 2019	2015
**Premature deaths or YLLs**	All causes	Adults	1.044	1.043 [1.026; 1.061]	1.059 [1.031; 1.088]	1.045 [1.029; 1.060]	1.045 [1.029; 1.060]			1.051 [1.029; 1.065]
		Infants			1.056 [1.026; 1.088]	1.04 [1.02; 1.07]				
	Lung cancer	Adults			1.106 [1.042; 1.174]			1.060 [1.020; 1.080]	1.112 [1.063; 1.129]	
**Attacks**	Asthma	Adults		1.044 [1.027; 1.062]	1.029 [1.013; 1.045]	1.029 [1.013; 1.045]				
		Children		1.039 [1.019; 1.059]		1.028 [1.006; 1.051]				
**Cases (incidence)**	Chronic bronchitis	Adults		1.098 [1.009; 1.194]	1.051 [1; 1.150]	1.117 [1.040; 1.189]				
**Cases (prevalence)**	Acute bronchitis	Children		1.306 [1.135; 1.502]	1.353 [1.095; 1.671]	1.080 [0.980; 1.190]				
**Hospital admissions**	CVD	All		1.012 [1.007; 1.019]		1.007 [1.001; 1.012]				
	RD	All		1.013 [1.001; 1.025]		1.014 [0.999; 1.029]				
**Hospital days**	CVD	All	1.009		1.007 [1.004; 1.009]					
	RD	All	1.015		1.008 [1.006; 1.011]					
**RADs**	All causes	Adults	1.105	1.094 [1.079; 1.109]	1.094 [1.080; 1.110]	1.034 [1.030; 1.038]				
**Work loss days**	All causes	Workers	1.105			1.033 [1.028; 1.038]				

Abbreviations: YLLs = Years of life lost. CVD = Cardio-vascular diseases. RD = Respiratory diseases. RADs = Restricted activity person-days.
[1] Age ranges of the population groups differ across AP-HRAs.

Table A 18 Mean excess relative risk of morbidity impacts across AP-HRAs, years expressed as per 10 µg/m^3 PM$_{10}$ and as a ratio in relation to the reference value (most recent STE, in bold). The ratio is calculated by dividing the AP-HRA value by the reference value (Switzerland 2021).

Type of impact	Outcome disease	Population group [1]	STE 1993	STE 1996	STE 2000&2005	STE 2010	EEA 2009-2018	FCAH 2010	GBD 1990-2019	CITIES 2015
Mean excess relative risk per 10 µg/m^3 PM$_{10}$										
**Attacks**	Asthma	Adults		0.044	0.029	**0.029**				
		Children		0.039		**0.028**				
**Cases (incidence)**	Chronic bronchitis	Adults		0.098	0.051	**0.117**				
**Cases (prevalence)**	Acute bronchitis	Children		0.306	0.353	**0.080**				
**Hospital admissions**	CVD	All		0.012		**0.007**				
	RD	All		0.013		**0.014**				
**Hospital days**	CVD	All	0.009		**0.007**					
	RD	All	0.015		**0.008**					
**RADs**	All causes	Adults	0.105	0.094	0.094	**0.034**				
**Work loss days**	All causes	Workers	0.105			**0.033**				
Ratio in relation to reference value (last STE) [2]										
**Attacks**	Asthma	Adults		1.52	1.00	**1**				
		Children		1.39		**1**				
**Cases (incidence)**	Chronic bronchitis	Adults		0.84	0.44	**1**				
**Cases (prevalence)**	Acute bronchitis	Children		3.82	4.41	**1**				
**Hospital admissions**	CVD	All		1.71		**1**				
	RD	All		0.93		**1**				
**Hospital days**	CVD	All	1.29		**1**					
	RD	All	1.87		**1**					
**RADs**	All causes	Adults	3.09	2.76	2.76	**1**				
**Work loss days**	All causes	Workers	3.18			**1**				

Abbreviations: YLLs = Years of life lost. CVD = Cardio-vascular diseases. RD = Respiratory diseases. RADs = Restricted activity person-days.

[1] Age ranges of the population groups differ across AP-HRAs.

[2] Examples for interpretation of the ratio: 1.1 = 1.1 times the ref. value = 10% higher. 2.0 = 2 times the ref. value = 100% higher. 0.4 = 0.4 times the ref. value = 60% lower.

Table A 19shows the CRFs in form of relative risks of the EEA and GBD before and after re-scaling using the equation above .

Table A 19 Relative risks before and after re-scaling from $PM_{2.5}$ to PM_{10} (Switzerland 2021).

Author	Type of impact	Outcome disease	Population group	Relative risk per 10µg/m^3	
				$PM_{2.5}$	PM_{10} (re-scaled)
**EEA**	Premature deaths	All causes	Adults	1.062 [1.04; 1.083]	1.045 [1.029; 1.06]
**CITIES**	Premature deaths	All causes	Adults	1.07 [1.04; 1.09]	1.051 [1.029; 1.065]
**GBD**	Premature deaths	Lung cancer	Adults	1.155 [1.086; 1.179]	1.112 [1.063; 1.129]

BASELINE HEALTH DATA AMONG POPULATION AT RISK

Table A 20 and Table A 21 show the age ranges of population at risk for mortality and morbidity outcomes respectively. Regarding morbidity, the previous STEs had higher numbers in the baseline health data than STE-2010 except for: bronchitis and restricted activity person-days in STE-2000; asthma and bronchitis in children in STE-1996; and hospital days due to cardiovascular diseases and restricted activity person-days for STE-1993, which report lower values than in STE-2010.

Furthermore, the main divergences can be found in the definition of adult and children for asthma and bronchitis. STE-2010 considered as adults people at the age 18 or older, while the previous STEs assumed 15 years old or older for asthma and 25 for bronchitis. Children were considered to be between 5 and 17 years old for STE-2010 and younger than 15 for previous STEs.

Table A 20 Mortality outcomes assessed and age groups assessed in the selected AP-HRAs (empty cells show non-assessed outcomes) (Switzerland 2021).

**Type of impact**	**Outcome disease**	**Population group**	**STE**	**STE**	**STE**	**STE**	**STE**	**EEA**	**FCAH**	**GBD** [1]	**WHO**	**CITIES**
			1993	1996	2000	2005	2010	2009-2018	2010	1990-2019	2012 & 2016	2015
**Premature deaths**	All causes	Adults	≥30	≥30	≥30		≥30	≥30		≥30	≥25	≥20
		Infants			<1		<1			<1	<5	
		Workers					30-85					
	Lung cancer	Adults			≥30				≥30	≥30	≥25	
**Working YLLs**	All causes	Adults			≥30		≥30					
		Infants					<1					
**YLLs**	All causes	Adults			≥30	≥30	≥30	≥30		≥30	≥25	≥20
		Infants			<1	<1	<1			<1	<5	

Abbreviations: YLLs = Years of life lost.

[1] The GBD data set contains assessments for more than 350 causes (diseases) and more than 50 age ranges. This table only shows diseases and ages that are comparable to other selected AP-HRAs.

Table A 21 Morbidity outcomes assessed and ages assessed in the selected AP-HRAs (empty cells show non-assessed outcomes) (Switzerland 2021).

Type of impact	Outcome disease	Population group	STE	STE	STE	STE	STE	GBD [1]	WHO
			1993	1996	2000	2005	2010	1990-2019	2012 & 2016
**Attacks**	Asthma	Adults		≥15	≥15	≥15	≥18		
**Attacks**	Asthma	Children		<15			5-17		
**Attacks (person-days)**	Asthma	Adults	≥15						
**Cases (incidence)**	Acute bronchitis	Children	<15						
**Cases (incidence)**	Chronic bronchitis	Adults		≥25	≥25	≥25	≥18		
**Cases (prevalence)**	Acute bronchitis	Children		<15	<15	<15	5-17		
**Cases (prevalence)**	Chronic bronchitis	Adults	≥25						
**DALYs**	All causes	Adults						≥30	≥25
**DALYs**	All causes	Infants						<1	<5
**Hospital admissions**	CDV	All		All			All		
**Hospital admissions**	RD	All		All			All		
**Hospital days**	CDV	All	All		All	All	All		
**Hospital days**	RD	All	All		All	All	All		
**Invalidity cases**	Chronic bronchitis	Adults	≥25						
**Medication intake (person-days)**	Asthma	Adults	≥15						
**RADs**	All causes	Adults	≥20	≥20	≥20	≥20	≥18		
**Symptom days**	RD	Children	<15						
**Symptom days**	RD	All	All						
**Work loss days**	All causes	Workers	≥15				≥15		
**YLDs**	All causes	All						All	

Abbreviations: DALYs = Disability-adjusted life years. RADs = Restricted activity person-days. YLDs = Years lived with disability. CVD = Cardio-vascular diseases. RD = Respiratory diseases.

[1] The GBD data set contains assessments for 364 diseases and 58 age ranges. This table only shows diseases and ages that are comparable to other AP-HRAs.

Table A 22 Morbidity baseline health data expressed as per 100,000 all-age persons and as a ratio in relation to the reference value (most recent STE). The ratio is calculated by dividing the AP-HRA value by the reference value (Switzerland 2021).

Type of impact	Outcome disease	Population group [1]	STE 1993	STE 1996	STE 2000	STE 2010	FCAH 2010	CITIES 2015
Baseline health data								
**Attacks**	Asthma	Adults		17,337	17,471	17,199		
		Children		5,766		42,817		
**Cases (incidence)**	Chronic bronchitis	Adults		502	248	319		
**Cases (prevalence)**	Acute bronchitis	Children		2,161	1,926	2,545		
**Hospital admissions**	CVD	All		2,471		1,862		
	RD	All		1,033		895		
**Hospital days**	CVD	All	10,894		17,936	17,897		
	RD	All	7,573		8,361	7,449		
**RADs**	All causes	Adults	400,134		251,241	1,556,074		
Ratio in relation to reference value (last STE) [2]								
**Attacks**	Asthma	Adults		1.01	1.02	1		
		Children		0.13		1		
**Cases (incidence)**	Chronic bronchitis	Adults		1.57	0.78	1		
**Cases (prevalence)**	Acute bronchitis	Children		0.85	0.76	1		
**Hospital admissions**	CVD	All		1.33		1		
	RD	All		1.15		1		
**Hospital days**	CVD	All	0.61		1	1		
	RD	All	1.02		1.12	1		
**RADs**	All causes	Adults	0.26		0.16	1		

Abbreviations: YLLs = Years of life lost. CVD = Cardio-vascular diseases. RD = Respiratory diseases. RADs = Restricted activity person-days.
Note: We did not found baseline health data in EEA, GBD and WHO.
[1] See age ranges of the population groups, which differ across AP-HRAs.
[2] Examples for interpretation of the ratio: 1.1 = 1.1 times the ref. value = 10% higher. 2.0 = 2 times the ref. value = 100% higher. 0.4 = 0.4 times the ref. value = 60% lower.

1. Devleesschauwer B, Havelaar AH, Maertens de Noordhout C, Haagsma JA, Praet N, Dorny P, et al. Calculating disability-adjusted life years to quantify burden of disease. International Journal of Public Health. 2014;59(3):565-9.

2. Online Database of State of Global Air. Data source: Global Burden of Disease Study 2019. [Internet]. Health Effects Institute (HEI). 2020 [cited 18/11/2020]. Available from: https://www.stateofglobalair.org/.

3. EEA-ETC/ATNI. Reference air quality maps 2005 and 2009. PM10, PM2.5, ozone and NO2spatial maps and population exposure.: European Topic Centre on Air pollution, transport, noiseand industrial pollution (ETC/ATNI) of the European Environment Agency (EEA). ETC/ATNI consortium partners:NILU –Norwegian Institute for Air Research, Aether Limited, Czech Hydrometeorological Institute (CHMI), EMISIA SA, Institut National de l'Environnement Industriel et des risques (INERIS), Universitat Autònoma de Barcelona (UAB), Umweltbundesamt GmbH (UBA-V), 4sfera Innova,Transport & Mobility Leuven NV (TML); 2020. Contract No.: 2020/1.

4. EEA. Briefing: Assessing the risks to health from air pollution European Environment Agency (EEA); 2018 [Available from: https://www.eea.europa.eu/themes/air/health-impacts-of-air-pollution/assessing-the-risks-to-health.

5. Zhang JJ, Hu W, Wei F, Wu G, Korn LR, Chapman RS. Children's respiratory morbidity prevalence in relation to air pollution in four Chinese cities. Environ Health Persp. 2002;110(9):961-7.

6. Hrubá F, Fabiánová E, Koppová K, Vandenberg JJ. Childhood respiratory symptoms, hospital admissions, and long-term exposure to airborne particulate matter. J Expo Anal Environ Epidemiol. 2001;11(1):33-40.

7. Dockery DW, Cunningham J, Damokosh AI, Neas LM, Spengler JD, Koutrakis P, et al. Health effects of acid aerosols on North American children: respiratory symptoms. Environ Health Persp. 1996;104(5):500-5.

8. Dockery DW, Speizer FE, Stram DO, Ware JH, Spengler JD, Ferris BG, Jr. Effects of inhalable particles on respiratory health of children. Am Rev Respir Dis. 1989;139(3):587-94.

9. Braun-Fahrländer C, Vuille JC, Sennhauser FH, Neu U, Künzle T, Grize L, et al. Respiratory health and long-term exposure to air pollutants in Swiss schoolchildren. SCARPOL Team. Swiss Study on Childhood Allergy and Respiratory Symptoms with Respect to Air Pollution, Climate and Pollen. Am J Respir Crit Care Med. 1997;155(3):1042-9.

10. Brauer M, Hoek G, Van Vliet P, Meliefste K, Fischer PH, Wijga A, et al. Air pollution from traffic and the development of respiratory infections and asthmatic and allergic symptoms in children. Am J Respir Crit Care Med. 2002;166(8):1092-8.

11. Hoek G, Pattenden S, Willers S, Antova T, Fabianova E, Braun-Fahrländer C, et al. PM10, and children's respiratory symptoms and lung function in the PATY study. Eur Respir J. 2012;40(3):538-47.

12. Miettinen OS. Proportion of disease caused or prevented by a given exposure, trait or intervention. Am J Epidemiol. 1974;99(5):325-32.

13. Levin ML. The occurrence of lung cancer in man. Acta Unio Int Contra Cancrum. 1953;9(3):531-41.

14. Lin C-K, Chen S-T. Estimation and application of population attributable fraction in ecological studies. Environmental Health. 2019;18(1):52.

15. Zapata-Diomedi B, Barendregt JJ, Veerman JL. Population attributable fraction: names, types and issues with incorrect interpretation of relative risks. British Journal of Sports Medicine. 2018;52(4):212.

16. ECOPLAN, SwissTPH. Aktualisierungstool externe Effekte des Verkehrs: Gesundheitskosten durch Luftverschmutzung (unpublished). 2013.

17. ARE, BAG, BFE, BUWAL. Externe Gesundheitskosten durch verkehrsbedingte Luftverschmutzung. Aktualisierung für das Jahr 2000.: Bundesamt für Raumentwicklung (ARE), Bundesamt für Gesundheit (BAG), Bundesamt für Energie (BFE), Bundesamt für Umwelt und Wirtschaft (BUWAL); 2004.

18. EEA-ETC/ACM. Quantifying the health impacts of ambient air pollution: methodology and input data ETC/ACM Technical Paper 2016/5 A consortium of European institutes under contract of the European Environment Agency (EEA) : RIVM Aether CHMI CSIC EMISIA INERIS NILU ÖKO-Institut ÖKO-Recherche PBL UAB UBA-V VITO 4Sfera 2016.

19. Murray CJL, Aravkin AY, Zheng P, Abbafati C, Abbas KM, Abbasi-Kangevari M, et al. Global burden of 87 risk factors in 204 countries and territories, 1990-2019: a systematic analysis for the Global Burden of Disease Study 2019 The Lancet. 2020;396(10258):1223-49.

20. Khomenko S, Cirach M, Pereira-Barboza E, Mueller N, Barrera-Gómez J, Rojas-Rueda D, et al. Premature mortality due to air pollution in European cities: a health impact assessment. The Lancet Planetary Health. 2021.

21. WHO. Ambient air pollution: A global assessment of exposure and burden of disease. World Health Organization (WHO); 2016.

22. ARE. Externe Effekte des Verkehrs 2010. Monetarisierung von Umwelt-, Unfall- und Gesundheitseffekte.: Bundesamt für Raumentwicklung (ARE); 2014.

23. EEA-ETC/ATNI. Health Risk Assessment of Air Pollutionin Europe. Methodology description and 2017 results.: European Topic Centre on Air pollution, transport, noise and industrial pollution (ETC/ATNI) of the European Environment Agency; 2020.

24. Castro A, Götschi T, Achermann B, Baltensperger U, Buchmann B, Felber Dietrich D, et al. Comparing the lung cancer burden of ambient particulate matter using scenarios of air quality standards versus acceptable risk levels. International Journal of Public Health. 2020;65(2):139-48.
