# Supplementary material for: Methods Matter: A Comparative Review of Health Risk Assessments for Ambient Air Pollution in Switzerland
Source: Public Health Rev. 2022 Apr 6;43:1604431. doi: 10.3389/phrs.2022.1604431 (PMC9020261; doi:10.3389/phrs.2022.1604431)
Supplement: Supplementary file 2 [file DataSheet1.PDF]

SUPPLEMENTARY MATERIAL 1: METHODS

LITERATURE REVIEW

We carried out a literature search in January 2021 to identify publications with health impact assessments of exposure to outdoor air pollution in Switzerland beyond the STEs. For this task, we used Google Search instead of research-specific searchers to find not only academic but also “grey” literature. Iteratively the following search terms was used: 1) Switzerland "air pollution" health burden assessment, 2) Switzerland "air pollution" deaths "years of life lost" mortality, 3) Switzerland "air pollution" "health impacts", 4) Switzerland "air pollution" "mortality". We examined the first 50 search results of each iteration.

Additionally, we carried out a specific search for studies using the three majoritarian Swiss local languages: German, French and Italian. Thus, we searched the following two word combinations: 1) Switzerland "air pollution” health and 2) Switzerland "air pollution” health city (i.e. same search terms just adding the word “city”). Translated into German, French and Italian: Schweiz Luftverschmutzung Gesundheit (Stadt), Suisse "pollution de l'air" santé (ville), Svizzera "inquinamento dell'aria" salute (città). We examined the first 20 search results of each search in local language.

Finally, we consulted the Swiss Literature Database on Air Pollution and Health (LUDOK in German) to confirm that we did not overlook a relevant air pollution health risk assessment (AP-HRA) (1).

Beyond the selected AP-HRAs, we excluded the following literature based on the inclusion criteria described. Out of the published STEs, we excluded the STE-2015 (2) and 2017 (3) because they only show transport-related external costs. Beyond the STEs, from the reviewed literature reviewed, we excluded some publications because they used results from other AP-HRAs such as the GBD (e.g. 4, 5) or made only future projections (e.g. 6). Some Swiss-designed projects were excluded because they assess transport-related (instead of all-source) emissions (e.g. 7, 8) or focused on some methodological aspects, .such as exposure-response models (e.g. 9, 10). Furthermore, although the EEA reports were selected for this study, we excluded those for 2009 and 2010 because they re-use GBD results. The above mentioned results for 2009 were compiled from the EEA report for 2018, which exceptionally included this new assessment of a past year (11).

In the literature review we additionally identified some local AP-HRAs at regional level, namely for the canton of Zurich (12, 13), the canton of Basel (14) and the agglomeration of Lausanne-Morges (15) (Table A 1). We excluded them because their small geographic scale does not enable a comparison with national scale AP-HRAs. Additionally, some of them re-use STE results (AP-HRAs in Zurich) or focus on comparing two years without specific result for each year (AP-HRA in Lausanne-Morges).

Table A 1 Regional AP-HRAs not selected for the comparison (Switzerland 2021).

**AP-HRA [1]**	**Year**	**Swiss area**	**Mortality outcomes**	**Morbidity outcomes**	**Pollutants**
BASEL	1996	Canton of Basel	• Lung cancer deaths		PM_{10}
LAUSANNE	2015	Agglomeration Lausanne-Morges	• Deaths	• Asthma attacks<br>• Bronchitis cases<br>• Hospital admissions<br>• Invalidity cases<br>• Restricted activity person-days<br>• Symptom days<br>• Work loss days	$PM_{2.5}$, NO_2
ZURICH	2005, 2010, 2015	Canton of Zurich	• Deaths	• Hospital admissions<br>• Bronchitis cases<br>• Restricted activity person-days<br>• Symptom days	PM_{10}, NO_2

[1] Sources: BASEL: Röösli, Künzli (14); LAUSANNE: Castro, Künzli (15); ZURICH: ECONCEPT (13).

Finally, we selected five AP-HRAs, which met the inclusion criteria and that were compared to the STEs (Figure A 1).

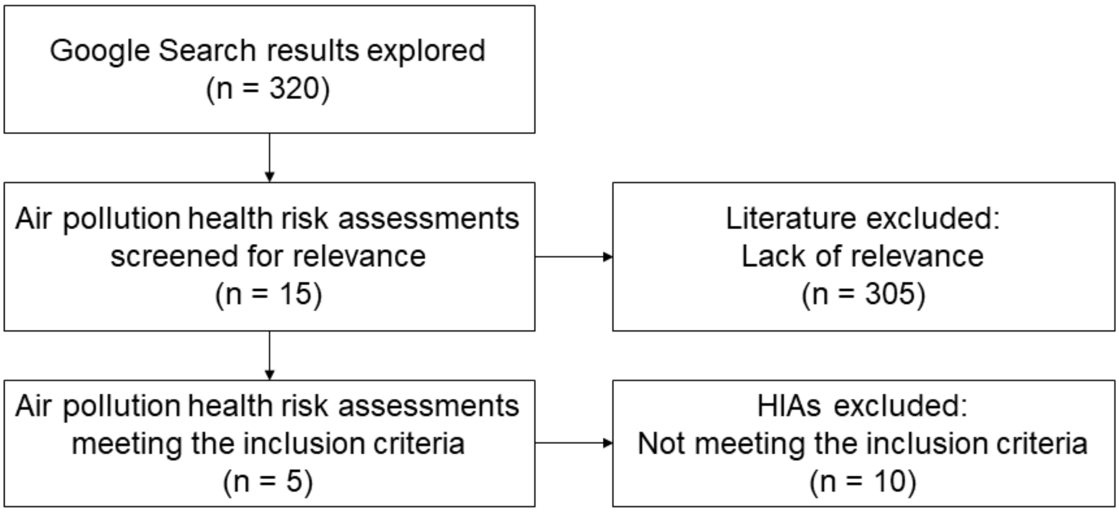

Figure A 1 Prisma flow chart of the literature review (Switzerland 2021).

DATA PROCESSING

Data filtering

We collected results and input data from all selected AP-HRAs, including all chosen pollutants, all years of analysis (whole time series), and the counterfactual scenarios. To show an overview of the assessed health impacts and the data heterogeneity, we limited the number of variables (pollutants, years of analysis, and outcomes), enabling a more targeted analysis, as follows.

For the overview of health impacts, we selected only one AP-HRA and year of analysis. We prioritized data from the last available STE over other AP-HRAs assuming that STEs have a better

knowledge of local circumstances. If no STE data were available, we selected data from other AP-HRA prioritizing the most recent ones.

For the heterogeneity of health impacts, we carried out a three-step filtering process. Firstly, we identified the pollutant with the highest attributed impacts pollutants by comparing AP-HRAs with more than one pollutant in their most recent overlapping year. In further steps, we focused on this pollutant. Secondly, we focused on the most relevant years of analysis. We included all STEs years, but in case of other AP-HRAs with time series, we selected only the first and the last year. We assumed that the first and last year capture the largest heterogeneity of data, given that air pollution concentration has decreased in Switzerland over the last decades (16). Thirdly, we removed the health outcomes that were assessed by only one AP-HRA or not assessed by a STE, since they do not allow comparability with STEs.

Normalization and re-scale

We normalized the absolute health impact from AP-HRAs by dividing by all-age population (per 100,000 persons). The normalization mitigates the effect of yearly variation due to changes in population and increases comparability across AP-HRAs. For population at national level, we used data from the Swiss Federal Office for Statistics (17), while for cities and agglomerations we used data from the AP-HRA to avoid discrepancies in the definition of the agglomeration boundaries.

Table A 2 and Table A 3 show the population data used for normalizing health impacts of the selected AP-HRAs. The national values are from Swiss Federal Office for Statistics (17), while the population for CITIES (ten largest urban areas) are from this AP-HRA. CITIES considered both cities and when available “greater cities”, which include the whole agglomeration beyond the city boundaries.

Table A 2 Population in Switzerland on January 1 (17) (Switzerland 2021).

Year	Population
**1990**	6,673,850
**1991**	6,757,188
**1992**	6,842,768
**1993**	6,907,959
**1994**	6,968,570
**1995**	7,019,019
**1996**	7,062,354
**1997**	7,081,346
**1998**	7,096,465
**1999**	7,123,537
**2000**	7,164,444
**2001**	7,197,638
**2002**	7,255,653
**2003**	7,313,853
**2004**	7,364,148
**2005**	7,415,102
**2006**	7,459,128
**2007**	7,508,739
**2008**	7,593,494
**2009**	7,701,856
**2010**	7,785,806
**2011**	7,870,134
**2012**	7,954,662
**2013**	8,039,060
**2014**	8,139,631
**2015**	8,237,666
**2016**	8,327,126
**2017**	8,419,550
**2018**	8,484,130
**2019**	8,544,527

Table A 3 Total (all-age) population in the ten largest Swiss urban areas in 2015 according to CITIES (18) (Switzerland 2021).

Category of the urban area	Name of the urban area	Population
**Greater City**	Zurich	618,300
**Greater City**	Geneva	368,188
**Greater City**	Basel	308,348
**Greater City**	Bern	215,216
**Greater City**	Lausanne	228,687
**City**	Winterthur	106,230
**City**	St. Gallen	74,024
**Greater City**	Luzern	152,531
**Greater City**	Lugano	81,929
**City**	Biel/Bienne	59,255
**Sum**	10 Swiss urban areas	2,212,708

Regarding the input data, conversions were required to make values comparable. Thus, we re-scaled concentration values from $PM_{2.5}$ into PM_{10} values assuming that $PM_{2.5}$ accounts for 73.5% of PM_{10}, i.e. by dividing $PM_{2.5}$ concentrations by a conversion factor of 0.735 (19). Additionally, we used this conversion factor to re-scale the CRF of the selected AP-HRAs. We converted the CRF

expressed in terms of $PM_{2.5}$ into PM_{10} exposure by applying a logarithmic transformation. Furthermore, most AP-HRAs express the counterfactual scenario as a single value, while some of them express it as a bound. We calculated the average of the lower and upper limits of these uniform distributions to make the values comparable with the others.

To compare PM_{10} with $PM_{2.5}$ values, we re-scaled $PM_{2.5}$ concentrations into a PM_{10} form using Equation A 1.

Equation A 1 Re-scale of $PM_{2.5}$ into PM_{10} concentration.

$$C_{PM10} = \frac{C_{PM2.5}}{CF}$$

C_{PM10} = PM_{10} concentration.
$C_{PM2.5}$ = $PM_{2.5}$ concentration.
CF = Conversion factor, i.e. proportion of $PM_{2.5}$ in PM_{10}, i.e. 73.5% (19, 20)

We compiled these data from the corresponding AP-HRAs. We re-scaled the relative risk coefficients of the selected studies expressed in terms of $PM_{2.5}$ exposure into PM_{10} exposure by applying Equation A 2. Thus, EEA used the following $PM_{2.5}$ relative risk for premature deaths in adults: 1.062 (95% CI: 1.04; 1.083). After re-scaling, the PM_{10} relative risk is 1.0452 (95% CI: 1.0292; 1.0604).

Equation A 2 Re-scale of relative risks from $PM_{2.5}$ to PM_{10}.

$$RR_{PM10} = e^{\ln(RR_{PM2.5})*CF}$$

RR_{PM10} = Relative risk for PM_{10} exposure
$RR_{PM2.5}$ = Relative risk for $PM_{2.5}$ exposure
CF = Conversion factor (proportion of $PM_{2.5}$ in PM_{10})

Ratio and reference value

To measure the heterogeneity of quantitative data (health impacts and input data) across AP-HRAs, we expressed the values of the AP-HRA as ratios in relation to a reference value. We assigned the reference value to the most recent STE of the selected AP-HRAs. If a certain health impact was not assessed in the most recent STE, the value of a previous STE was selected as reference value.

We calculated the ratios by dividing the value of the AP-HRA by the reference value. Thus, ratios less than 1 show that the AP-HRA value is lower than the reference value, while ratios higher than 1 show AP-HRA values higher than reference value. Exceptionally, for CRF, we subtracted one from both the numerator and denominator before dividing, i.e. we calculated the ratio of the excess relative risk.

Data preparation and assumptions

Collected data required minor edits as follows. For STE-2000 and STE-2005, we calculated the YLLs in adults and infants based on the total number of YLLs as follows. The AP-HRAs states that 96% of the total number refers to adults and 4% to infants. For AP-HRAs with results at city level, we summed up the health impacts of the cities. For STE-2005, we calculated the reference value by multiplying the value in 2000 by 1.0289 because STE-2005 only indicates that the value is 2.89% higher than in STE-2000. For WHO-2012, we assigned the mortality to infants and the sum of the rest of disease-specific health impacts to adults, as described in the report.

Furthermore, in case of unclear information, we had to make assumptions as follows. Given that the GBD dataset categorizes lung cancer as “tracheal, bronchus, and lung cancer”, we assumed that the value is comparable with lung cancer values provided in other AP-HRAs. Concerning the age ranges that correspond to the population groups (adults vs. children vs. infants) we made multiple assumptions. STE-2005 (short update of STE-2000) does not provide the age of the YLLs; thus, we assumed the age of STE 2000. STE-1993 only provided the population group for premature deaths, acute bronchitis in children and symptom days as well as the age for acute bronchitis in children; we deduced the missing information based on later STE studies (if available). Otherwise, we made further assumptions. Thus, we deduced the age of incidence of chronic bronchitis and invalidity cases in adults for prevalence. We deduced the age of bronchitis in children for symptom days and the age of asthma attacks for days of medication intake of this disease. Additionally, we deduced that cases of acute bronchitis in children refer to prevalence instead of incidence based on next STE.

WHO-2016 presents specific DALYs for lower respiratory infections in children younger than 5 years old, while this value was not available for premature deaths and YLLs. Therefore, we assumed that it this value was zero, i.e. the whole number of premature deaths and YLLs refer to people at the age of 25 years or older.

REFERENCES

1. Swiss TPH. Swiss Literature Database on Air Pollution and Health (LUDOK). 2021.

2. ARE. Externe Effekte des Verkehrs 2015. Aktualisierung der Berechnungen von Umwelt, Unfall- und Gesundheitseffekten des Strassen-, Schienen-, Luft- und Schiffsverkehrs 2010 bis 2015. Bundesamt für Raumentwicklung (ARE); 2019.

3. ARE. Externe Kosten und Nutzen des Verkehrs in der Schweiz. Strassen-, Schienen-, Luft- und Schiffsverkehr 2017. Bundesamt für Raumentwicklung (ARE); 2020.

4. World Bank, IHME. The Cost of Air Pollution: Strengthening the Economic Case for Action. Washington, DC.: World Bank and Institute for Health Metrics and Evaluation (IHME); 2016.

5. OECD. The Cost of Air Pollution: Health Impacts of Road Transport. 2014.

6. Foreman KJ, Marquez N, Dolgert A, Fukutaki K, Fullman N, McGaughey M, et al. Forecasting life expectancy, years of life lost, and all-cause and cause-specific mortality for 250 causes of death: reference and alternative scenarios for 2016–40 for 195 countries and territories. The Lancet. 2018;392(10159):2052-90.

7. Vienneau D, Perez L, Schindler C, Lieb C, Sommer H, Probst-Hensch N, et al. Years of life lost and morbidity cases attributable to transportation noise and air pollution: A comparative health risk assessment for Switzerland in 2010. International Journal of Hygiene and Environmental Health. 2015;218(6):514-21.

8. Perez L, Trüeb S, Cowie H, Keuken MP, Mudu P, Ragettli MS, et al. Transport-related measures to mitigate climate change in Basel, Switzerland: A health-effectiveness comparison study. Environment International. 2015;85:111-9.

9. Röösli M, Künzli N, Braun-Fahrländer C, Egger M. Years of life lost attributable to air pollution in Switzerland: dynamic exposure–response model. International Journal of Epidemiology. 2005;34(5):1029-35.

10. Röösli M. Years of Life Lost Due to Air Pollution in Switzerland: A Dynamic Exposure-Response Model. In: Preedy VR, Watson RR, editors. Handbook of Disease Burdens and Quality of Life Measures. New York, NY: Springer New York; 2010. p. 685-99.

11. EEA. Air quality in Europe — 2020 report. European Environmental Agency (EEA); 2020. Contract No.: No 11/2020.

12. ECONCEPT. Die Kosten von Luftverschmutzung und Treibhausgasemissionen im Kanton Zürich 2005. AWEL; 2006.

13. ECONCEPT. Die Kosten der Luftverschmutzung 2005 bis 2015. Amt für Abfall, Wasser, Energie und Luft (AWEL), Stadt Zürich, Stadt Winterthur; 2018.

14. Röösli M, Künzli N, Schindler C, Theis G, Oglesby L, Mathys P, et al. Single Pollutant Versus Surrogate Measure Approaches: Do Single Pollutant Risk Assessments Underestimate the Impact of Air Pollution on Lung Cancer Risk? Journal of Occupational and Environmental Medicine. 2003;45(7).

15. Castro A, Künzli N, Götschi T. Health benefits of a reduction of PM10 and NO2 exposure after implementing a clean air plan in the Agglomeration Lausanne-Morges. International Journal of Hygiene and Environmental Health. 2017;220(5):829-39.

16. FOEN. Luftqualität 2019. Messresultate des Nationalen Beobachtungsnetzes für Luftfremdstoffe (NABEL). . Bundesamt für Umwelt (BAFU, FOEN in English); 2020.

17. Bilanz der ständigen Wohnbevölkerung, 1861-2019 [Internet]. Federal Statistical Office (BFS, in German). 2020. Available from: https://www.bfs.admin.ch/bfs/de/home/statistiken/bevoelkerung/stand-entwicklung/bevoelkerung.assetdetail.13707405.html.

18. Khomenko S, Cirach M, Pereira-Barboza E, Mueller N, Barrera-Gómez J, Rojas-Rueda D, et al. Premature mortality due to air pollution in European cities: a health impact assessment. The Lancet Planetary Health. 2021.

19. Castro A, Götschi T, Achermann B, Baltensperger U, Buchmann B, Felber Dietrich D, et al. Comparing the lung cancer burden of ambient particulate matter using scenarios of air quality standards versus acceptable risk levels. International Journal of Public Health. 2020;65(2):139-48.

20. FOEN. Air pollution concentration data for Switzerland. E-mail communication with Rudolf Weber (Federal Office for the Environment). Unpublished work. 2019.
